# Degradation, Promoter Recruitment and Transactivation Mediated by the Extreme N-Terminus of MHC Class II Transactivator CIITA Isoform III

**DOI:** 10.1371/journal.pone.0148753

**Published:** 2016-02-12

**Authors:** Yves B. Beaulieu, Jorge A. Leon Machado, Sylvain Ethier, Luc Gaudreau, Viktor Steimle

**Affiliations:** Département de biologie, Université de Sherbrooke, Sherbrooke, Qc, Canada; Vita-Salute San Raffaele University School of Medicine, ITALY

## Abstract

Multiple relationships between ubiquitin-proteasome mediated protein turnover and transcriptional activation have been well documented, but the underlying mechanisms are still poorly understood. One way to induce degradation is via ubiquitination of the N-terminal α-amino group of proteins. The major histocompatibility complex (MHC) class II transactivator CIITA is the master regulator of MHC class II gene expression and we found earlier that CIITA is a short-lived protein. Using stable and transient transfections of different CIITA constructs into HEK-293 and HeLa cell lines, we show here that the extreme N-terminal end of CIITA isoform III induces both rapid degradation and transactivation. It is essential that this sequence resides at the N-terminal end of the protein since blocking of the N-terminal end with an epitope-tag stabilizes the protein and reduces transactivation potential. The first ten amino acids of CIITA isoform III act as a portable degron and transactivation sequence when transferred as N-terminal extension to truncated CIITA constructs and are also able to destabilize a heterologous protein. The same is observed with the N-terminal ends of several known N-terminal ubiquitination substrates, such as Id2, Cdt1 and MyoD. Arginine and proline residues within the N-terminal ends contribute to rapid turnover. The N-terminal end of CIITA isoform III is responsible for efficient *in vivo* recruitment to the HLA-DRA promoter and increased interaction with components of the transcription machinery, such as TBP, p300, p400/Domino, the 19S ATPase S8, and the MHC-II promoter binding complex RFX. These experiments reveal a novel function of free N-terminal ends of proteins in degradation-dependent transcriptional activation.

## Introduction

Gene expression and the ubiquitin-proteasome system (UPS) are connected via several different mechanisms and both proteolytic and non-proteolytic functions link the UPS and transcription [1–3]. The 26S proteasome is composed of a 20S proteolytic complex and a 19S regulatory cap, and genome-wide protein-chromatin association studies in *S*. *cerevisiae* revealed that both subunits interact with a large number of genes, correlating with transcription. However, there was only a partial overlap of binding patterns and many genes interact preferentially with one or the other complex [4, 5]. On the other hand, the 26S proteasome associates also with a large number of genes *in vivo* and inhibition of proteolysis interferes with the expression of many genes [4–8]. Furthermore, many transcriptional activators, especially those with acidic activation domains (AADs) are short-lived proteins and transcriptional activation domains (TADs) often overlap closely with protein destabilizing sequence motifs (degrons) [3].

Ubiquitination is not only involved in transcription initiation. Turnover of the yeast transcription factor Gal4 was shown to be important for the co-transcriptional processing of Gal4-dependent mRNAs [9], while mono-ubiquitination of an artificial activator containing a VP16 AAD increased its interaction with the positive transcription elongation factor (P-TEFB) and stimulated transcriptional elongation rather then initiation [10].

Ubiquitin is usually linked covalently at its C-terminus to the ε-amino group of an internal lysine residue in the target protein, but the free α-amino group of the N-terminal end of a protein can also serve as a substrate for fusion with ubiquitin [11–14]. Apart from inducing protein degradation, the functional consequences of N-terminal ubiquitination (NTU) are unknown at present.

MHC class II (MHC-II) molecules serve as antigen presentation molecules for CD4-positive T cells and thus play a crucial role for the adaptive immune response. MHC-II genes show complex transcriptional regulation with cell-type specific constitutive and inducible expression patterns [15, 16]. The MHC-II transactivator CIITA is the master regulator of MHC-II gene transcription [16–19]. CIITA does not bind to DNA directly but is rather recruited to MHC-II gene promoters via protein-protein interactions with the ubiquitously expressed RFX, CREB and NF-Y complexes. These bind to conserved MHC-II promoter elements in an enhanceosome-like fashion, but are unable to activate MHC-II genes in the absence of CIITA [15, 16, 19]. CIITA is thus the main activator of MHC-II gene transcription and a close quantitative correlation exists between CIITA and MHC-II gene expression [20].

**Fig 1:**
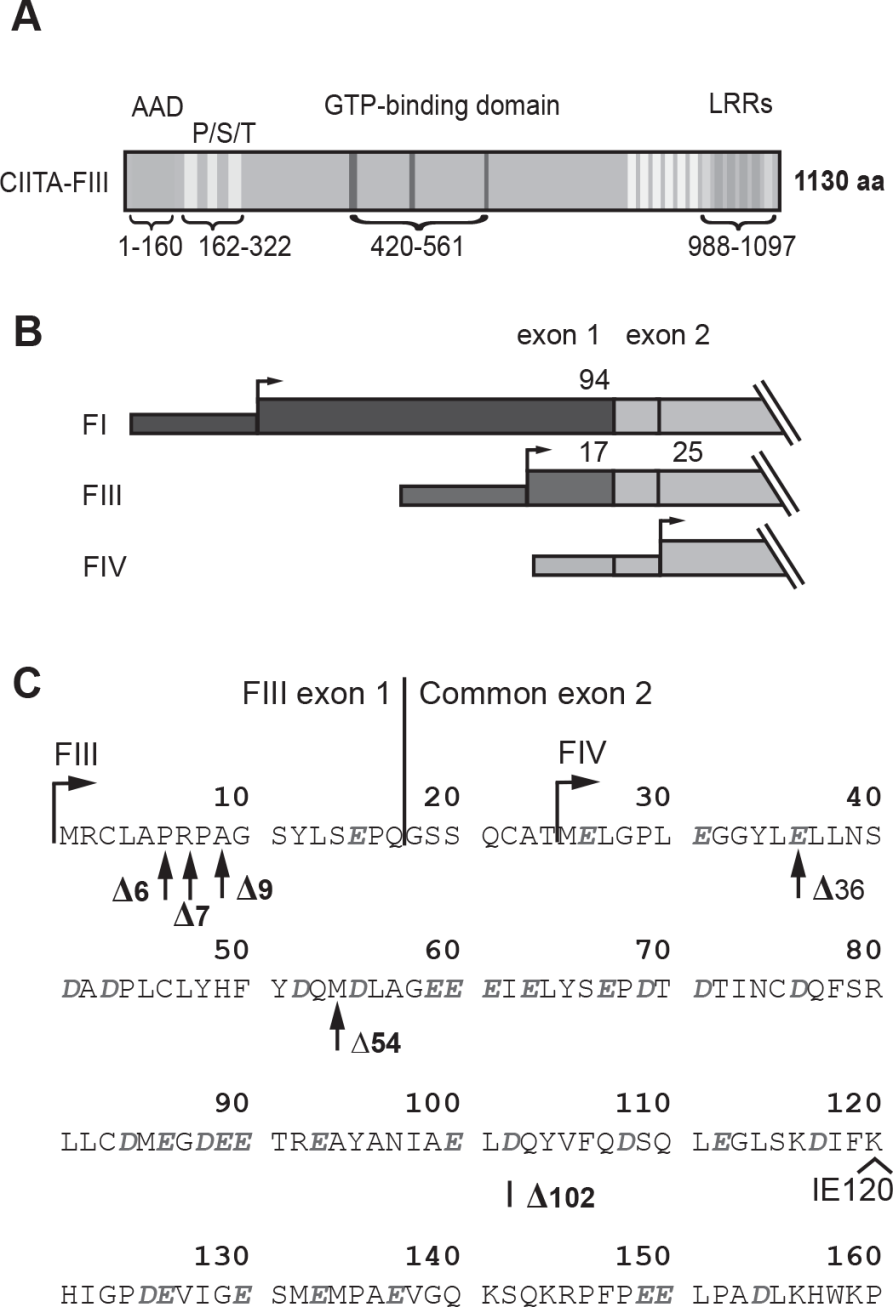
Domain structure of naturally occurring isoforms of CIITA and N-terminal deletion mutants used in this study. **A)** Domain structure of CIITA; numbering refers to CIITA-FIII (K_120_ isoform). **B)** N-terminal ends of naturally occurring forms CIITA-FI, -FIII and–FIV generated through alternative promoter usage. **C)** Amino acid sequence of the acidic activation domain (AAD) of CIITA-FIII (single letter code). Acidic amino acids are shown in italics. The N-terminal ends of CIITA-FIV and of N-terminal deletion mutants are indicated by arrows, and replacement of K_120_ by IE in the I_120_E_121_ isoform is also indicated.

CIITA contains an N-terminal AAD, followed by a region rich in proline, serine and threonine (P/S/T region), a central GTP-binding domain and a C-terminal leucine rich repeat domain (LRRs, Fig 1A) [17, 21]. Alternative promoter usage leads to the generation of three isoforms of CIITA (CIITA-FI, -FIII, and -FIV), differing only in their N-terminal ends [22] (Fig 1B). The initiation codon of CIITA-FIV is situated in the common exon 2, and thus CIITA-FIV (1106 amino acids) starts at Met_25_ of CIITA-FIII (1130 amino acids; Fig 1B and 1C). Exons 1 of CIITA-FI (1208 amino acids) and FIII have their own in-frame initiation codons leading to two different N-terminal ends of respectively 94 or 17 amino acids N-terminal to the splice junction of the common exon 2 (Fig 1B).

The N-terminal AAD of CIITA appears to function in a similar manner as the well-characterized AAD of VP16, interacting with components of the basal transcription machinery and co-activators such as TBP-associated factors (TAFs), p300/CBP and PCAF [19, 21]. Both the VP16 AAD and CIITA stimulate not only transcriptional initiation, but also elongation by recruiting P-TEFB, and in both cases a synthetic fusion of the activation domain with a non-polymerizable mono-ubiquitin chain led to increased interaction with the CycT1 subunit of P-TEFB [10, 23].

We found earlier that CIITA-FIII is rapidly turned over by the UPS, showing a half-life of about 30–40 minutes and that regions within the P/S/T domain and within the first 100 amino acids of the AAD mediate degradation [24]. Furthermore, N-terminal fusions with epitope-tags greatly stabilized CIITA [24]. Greer and colleagues reported that mono-ubiquitination of CIITA stimulates its transactivation potential in a nondegradative manner [25]. However, these experiments were carried out with N-terminally tagged forms of CIITA-FIII that, in agreement with our results [24], were strongly stabilized showing a half-life of over 4 h [25].

Structure/function analysis of CIITA has so far predominantly relied on the use of N-terminally epitope-tagged versions of CIITA. Here we analyzed the contribution of the N-terminal ends of naturally occurring forms and engineered (but untagged) mutants of CIITA to the activation of endogenous MHC-II genes. The different naturally occurring forms of CIITA and several engineered mutants all show an inverse correlation between protein expression levels and transactivation potential, with CIITA-FIII being the lowest expressed and transcriptionally most active form. Low protein expression levels are due to increased turnover. Even very short N-terminal deletions or point mutations within the N-terminal end of CIITA-FIII stabilize the protein and lead to a loss of transactivation potential. Furthermore, the first ten amino acids of CIITA-FIII and of several previously characterized substrates of the NTU pathway confer increased turnover and transactivation to truncated and stabilized forms of CIITA. We also show that even a very stable protein such as EYFP is destabilized by the N-terminal addition of the first ten amino acids of CIITA-FIII. Thus N-terminal ends of NTU proteins can function as portable degrons and transactivation domains. Proline and arginine residues within these N-terminal ends contribute to rapid turnover. Finally we provide evidence that CIITA-FIII is recruited to the HLA-DRA promoter more efficiently compared to the more stable forms, and interacts more strongly with components of the transcription machinery and MHC-II promoter binding proteins.

## Materials and Methods

### Cell culture and transfections

The CIITA-negative cell line HEK293-EBNA (Invitrogen R620-07), HeLa cells (ATCC CCL-2) and MEF-p300^fl/fl^-CBP^fl/fl^ [26], a kind gift of Dr. K. Ge, were grown in DMEM supplemented with 10% fetal calf serum. HEK293-EBNA cells were transfected by CaPO_4_ precipitation. Stable transfectants were selected for at least two weeks with hygromycin B (200 μg/ml, Calbiochem) and analyzed in bulk. HeLa and MEF-p300^fl/fl^-CBP^fl/fl^ cells were transiently transfected with PEI-max (Polysciences Inc.). Cells were analyzed 2 or 3 days after transfection.

### Expression vectors and cDNA expression constructs

cDNA constructs were expressed using EBV episomal expression vector EBS-NPL [27]. EBS-constructs for CIITA-FIII, ∆36, ∆54, ∆102 and Myc6-FIII have been described [24]. These and the other CIITA forms used here were generated on the backbone of a CIITA-FIII (I_120_E_121_) cDNA lacking the 3’UTR [28]. CIITA forms with different N-terminal ends were generated by exchanges with restriction fragments from other CIITA cDNA clones, by PCR mutagenesis, or via oligonucleotide adaptors. For the generation of CIITA-E163 constructs a DNA fragment coding for the EGFP ORF was inserted at amino acid position 163 of CIITA-FIII. The various CIITA-E163 constructs with different N-terminal ends were constructed as described above. All constructs were expressed in EBS-NPL. ùConstructs with N-terminal ten amino acids extensions are based on pEYFP-N1 and pEYFP-Nuc (Clontech). All constructs were verified by sequencing. EBS-PL-tdtomato was obtained from D. Garcin [29].

### CIITA-specific antisera

Rabbit antiserum K5 has been described [30]. Serum K5 was raised against an *E*.*coli* expressed N-terminal fragment of CIITA (amino acids 25 to 408 of CIITA-FIII). Serum K22 was raised against an *E*.*coli* expressed C-terminal fragment of CIITA (amino acids 335 to 1130 of CIITA-FIII). Both sera were absorbed on HeLa acetone powder. Serum K22 recognizes all CIITA forms used in this study equally well, but serum K5 showed higher sensitivity and was used for most experiments. Comparison of sera K5 and K22 on full length and truncated forms of CIITA showed that serum K5 recognizes mainly antigenic determinants within the AAD. CIITA-FI, -FIII, -FIV and ∆36 are all recognized equally well, while ∆54 is underestimated about twofold and ∆102 about eightfold compared to CIITA-FIII in western blots when using serum K5 ([30]; and data not shown).

### Other antibodies and antisera

mAb RW144 [31] recognizing both p300 and p400/Domino [32], was kindly provided by Dr. DM Livingston. The p300 specific mAb RW128 [31] was obtained from Novus Biologicals. The rabbit antisera specific for TBP (sc-273), RFX5 (#100-401-194), and the 19S ATPase S8 (A300-791A) were from Santa Cruz Biotechnology, Rockland Immunochemicals, and Bethyl Laboratories, respectively. The Hsp90 specific rabbit antiserum [33] was kindly provided by Dr RM Tanguay.

### Flow cytometry analysis

Flow cytometry analysis of live cells was performed on a FACSCalibur instrument (Becton Dickinson). Cells were stained with anti-human HLA-DR coupled to Quantum Red (Clone HK14, Sigma), or with the HLA-DR specific antibody D1.12 [34] (kindly provided by Dr RS Accolla) followed by staining with goat anti-mouse IgG F(ab’)2 coupled to Alexa-647 (Molecular Probes).

For transient transfections in HeLa and MEF-p300^fl/fl^-CBP^fl/fl^ cells, the cells were co-transfected with a red-fluorescent marker gene (d2tomato; a destabilized version of tdtomato [35]) and EGFP or EYFP fluorescence (FL1) and HLA-DR expression (FL4) were analyzed on d2tomato-positive cells (FL3).

### Cycloheximide (CHX) treatment, protein extraction, co-immunoprecipitation, and quantitative western blotting

HEK293-EBNA cells were incubated with 200 μg/ml of CHX (Sigma) and HeLa cells were incubated with 40 μg/ml CHX for the indicated times. Freeze-thaw protein extraction, SDS-PAGE, semi-dry blotting and western blotting were carried out as described [24, 27]. Unless indicated otherwise, 40μg of protein extract was separated on 6% gels. For co-immunoprecipitation assays, protein extract was immunoprecipitated with CIITA-specific antiserum K5 and protein A-sepharose, separated by SDS-PAGE, and proteins were transferred by wet blot. Primary antibodies were detected using the Supersignal West Femto kit (Pierce), and exposure to x-ray films, with the exception of S8, which was detected using Reliablot reagent (Bethyl Labs). Films were scanned on a GS-800 calibrated densitometer (BioRad) and quantified using the *Quantity One* software (BioRad).

### ChIP assays

ChIP assays were performed as described [36] with the CIITA-specific antiserum K22. As a control, ChIP was performed without antibody. HLA-DRA promoter fragments were amplified with primer pair F1/R1 (S1 Table) and analyzed by QPCR.

### Knock out experiments using a lentiviral Cre expression vector

The cre expressing lentiviral vector (Puro.Cre) was obtained from Addgene (#17408, [37]). Recombinant lentivirus was produced in Hek293T cells using psPAX2 packaging and pMD2.G VSV G envelope vectors according to protocols from the Trono laboratory (http://tronolab.epfl.ch/). MEF-p300^fl/fl^-CBP^fl/fl^ cells were infected with fresh supernatant of Cre expressing virus or an empty control vector. The next day cells were transfected with PEI-max and analyzed three days after transfection.

### Gene expression and QPCR analysis

Total RNA was isolated (AbsolutelyRNA kit; Stratagene) and first-strand cDNA generated with random nonamers using Expand RT (Roche). QPCR was performed using Power SYBR® Green PCR Master Mix reagents (ABI) on an ABI 7500 instrument. Samples were quantified with a standard curve and were normalized for HPRT expression. Primer sequences are shown in S1 Table.

### Statistical Analysis

Statistical significance between the different treatments was evaluated by using a Student t-test assuming unequal variances, allowing α = 0.05. An F-test was performed to prove unequal variances between the values from technical replicates.

## Results

### Steady state levels and turnover rates of different isoforms of CIITA are inversely correlated to transcriptional activity

Three forms of CIITA are generated via the usage of alternative promoters, (CIITA-FI, -FIII, -FIV; Fig 1B). During our initial cloning of CIITA isoform III, we also noted the existence of a fully functional variant form of CIITA, where amino acid position K_120_ (numbering refers to CIITA-FIII, Accession NM_000246) is replaced by I_120_E_121_ through alternative splicing (Fig 1C, S1 Fig; Accession NM_001286402) [17]. In the Burkitt lymphoma cell line Raji, which expresses mostly CIITA-FIII, both forms are expressed in similar amounts (data not shown). Unless specified, all constructs used here are I_120_E_121_ forms of CIITA.

**Fig 2:**
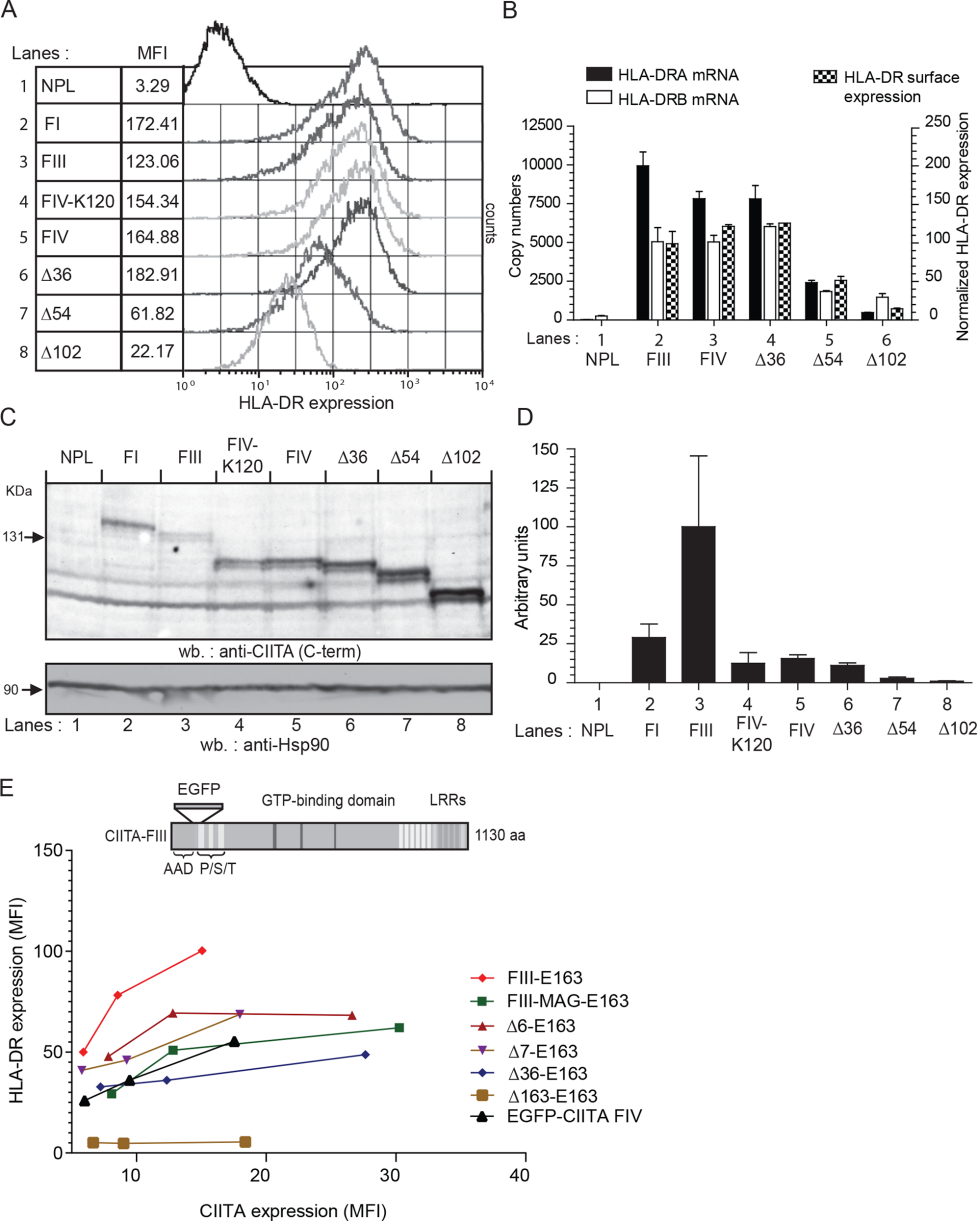
Steady-state protein expression levels and relative MHC-II activation potential of naturally occurring and engineered N-terminal variants of CIITA. **A)** Stable transfectants of indicated CIITA-variants were generated in HEK293-EBNA cells and bulk transfectants analyzed for cell surface HLA-DR expression by flow cytometry. NPL indicates cells transfected with empty expression vector (EBS-NPL). Mean fluorescence intensities (MFI) for the different transfectants are shown. **B)**. HLA-DRA and HLA-DRB expression of mature (spliced) mRNA was determined by RT-QPCR. mRNA expression levels (scale on left) were determined with linearized plasmid DNA as standards. HLA-DR expression of the cells used for RNA isolation is also shown (scale on right). Expression levels were normalized and expression of CIITA-FIII was set at 100 (values and standard errors from 2 independent experiments). **C)** Western blot analysis for CIITA protein expression of cells shown in (A). CIITA was detected with serum K22 (top). The blot was stripped and re-probed with an Hsp90-specific antiserum as loading control (bottom). **D)** Determination of relative transactivation potential of different CIITA forms based on MFI values of cell surface HLA-DR expression compared to CIITA protein expression levels. The activity of CIITA-FIII was set at 100. Values are derived from duplicates of flow cytometry analysis, protein extraction and western blotting. **E)** HeLa cells were transiently transfected with the indicated CIITA-E163 constructs and analyzed three days after transfection by flow cytometry for EGFP (FL1) and HLA-DR (FL4) expression. For each construct three different concentrations of plasmid were transfected: 0.05, 0.1, and 0.2 μg/well in 12-well plates. Cells were co-transfected with 0.2 μg/well d2tomato as a transfection marker and total DNA amount was completed to 0.7 μg DNA/well with empty expression vector. EGFP and HLA-DR expression were quantified on tomato-positive cells. All transfections were carried out in duplicate. The symbols were connected by lines only to improve clarity.

We first compared the transactivation potential of the naturally occurring CIITA forms (FI, FIII, FIV and FIV-K120) and of N-terminal deletion mutants CIITA-∆36, -∆54 and -∆102 (Figs 1C and 2). All constructs were expressed in HEK293-EBNA cells, which are negative for CIITA and MHC-II gene expression [24, 27, 30]. Stable transfectants were analyzed by flow cytometry for HLA-DR cell surface expression (Fig 2A). HLA-DR expression levels of transfectants of all natural CIITA isoforms and of CIITA-∆36 were very similar, while CIITA-∆54 showed a reduced HLA-DR cell surface expression, which was even lower for CIITA-∆102 (Fig 2A).

HLA-DR molecules are αβ-heterodimers and expression of the corresponding HLA-DRA and HLA-DRB genes was determined by RT-QPCR. HLA-DRB mRNA expression levels, which were lower than HLA-DRA copy numbers, correlated very closely with HLA-DR cell surface expression (Fig 2B). In addition, considerably higher HLA-DR expression levels were observed at intermediate time points of hygromycin B selection indicating that expression levels in stable CIITA-transfected HEK293-EBNA cells are not at saturation levels (S6A Fig). Thus HLA-DR cell surface expression is a reliable measure for the transactivation potency of CIITA.

We next analyzed CIITA steady-state protein expression levels in these transfectants by western blotting with the CIITA-specific antiserum K22, which was raised against amino acids 335–1130 and thus recognizes all CIITA forms used here equally well (Fig 2C). CIITA-FIII was expressed at the lowest levels, while CIITA-FI and all truncated forms, including the naturally occurring FIV forms showed higher steady-state levels. Expression levels increased incrementally with the progressive shortening of the N-terminal AAD, with ∆36, ∆54, and ∆102 each showing a higher expression than the next longer form (Fig 2C).

Thus CIITA-FIII activated MHC-II expression as well as the other forms (FI, FIV, and ∆36) despite the fact that it was expressed at a much lower level. Therefore the transactivation potential of different forms of CIITA was inversely correlated to protein expression levels and CIITA-FIII was the relatively most active form. Western blotting carried out under conditions that permitted reliable quantification of protein expression levels (see S2 Fig) revealed that CIITA-FIII shows at least a 5-fold higher relative transactivation potential compared to the other naturally occurring forms of CIITA and ∆36 (Fig 2D). Comparable results were also obtained in transient transfections (S3 Fig).

In order to demonstrate the higher activation potential of CIITA FIII more directly, we generated EGFP-labeled CIITA constructs. Since we wanted to compare different N-terminal ends of CIITA, it was obviously impossible to use EGFP as an N-terminal tag and C-terminal tags have in our hands always led to nonfunctional CIITA protein (unpublished results). We therefore inserted EGFP in position 163 of CIITA FIII (CIITA-E163; Fig 2E), which is at the C-terminal end of the N-terminal acidic domain of CIITA (see Fig 1). This permitted us to compare CIITA constructs with different N-terminal ends. For these experiments we carried out transient transfections with different doses of DNA in HeLa cells and analyzed EGFP and HLA-DR expression via flow cytometry (Fig 2E). The previous experiments had suggested that the region responsible for increased turnover and transactivation potential was localized within the first 25 amino acids of CIITA-FIII and possibly within the extreme N-terminal end. We therefore generated two additional N-terminal deletion mutants, in which the first 5 or 6 amino acids of CIITA-FIII were removed (∆6, ∆7). Furthermore we included a construct, in which amino acid positions 2 and 3 (Arg_2_, Cys_3_) were replaced by alanine and glycine (FIII-MAG-E163). We also tested an N-terminal fusion of EGFP with CIITA (EGFP-CIITA). A construct in which the first 163 amino acids of CIITA were completely deleted (∆163-E163) was used as a negative control for HLA-DR activation. The results suggest that CIITA-FIII-E163 activates MHC-II genes more strongly while showing a lower EGFP expression compared to the other constructs (Fig 2E). These experiments confirmed our previous observations in stable HEK 293EBNA transfectants and suggested that the extreme N-terminal end of CIITA is necessary for the observed effect. They also show that the effect is not dependent on a particular cell type or transfection method.

The peptide sequence encoded by exon 1 of CIITA-FI shows homology to caspase activation and recruitment domains (CARD) and Nickerson and colleagues reported that when equal amounts of DNA are transfected transiently, Flag-CIITA-FI shows a higher transcriptional activity compared to Flag-CIITA-FIII [38], but protein expression levels of CIITA were not analyzed. However, when CIITA protein expression levels are taken into account, CIITA-FI is actually transcriptionally less active than CIITA-FIII (Fig 2).

Many degradation signals are dependent on serine phosphorylation, and Drozina and colleagues as well as Bhat and colleagues showed that phosphorylation of S_357_ in CIITA-FI, which corresponds to S_280_ in CIITA-FIII, is involved in ubiquitination and transactivation [23, 39]. Phosphorylation leads to the appearance of a double band for CIITA in western blots and we observed double bands for all our constructs, including ∆36 constructs with N-terminal ends from other proteins. We treated protein extracts of cells expressing CIITA-FIII, -FIV, -∆36, -∆54 and -∆102 with λ-phosphatase and found that in all cases the upper of the two bands disappeared, suggesting that all forms tested here are partially phosphorylated (S4 Fig). The fact that the double band is present for all naturally occurring and engineered mutants analyzed here suggests that the observed stabilization of natural variants and mutants is not related to changes in phosphorylation levels.

### Rapid protein turnover of CIITA-FIII depends on the integrity of its N-terminal end

**Fig 3:**
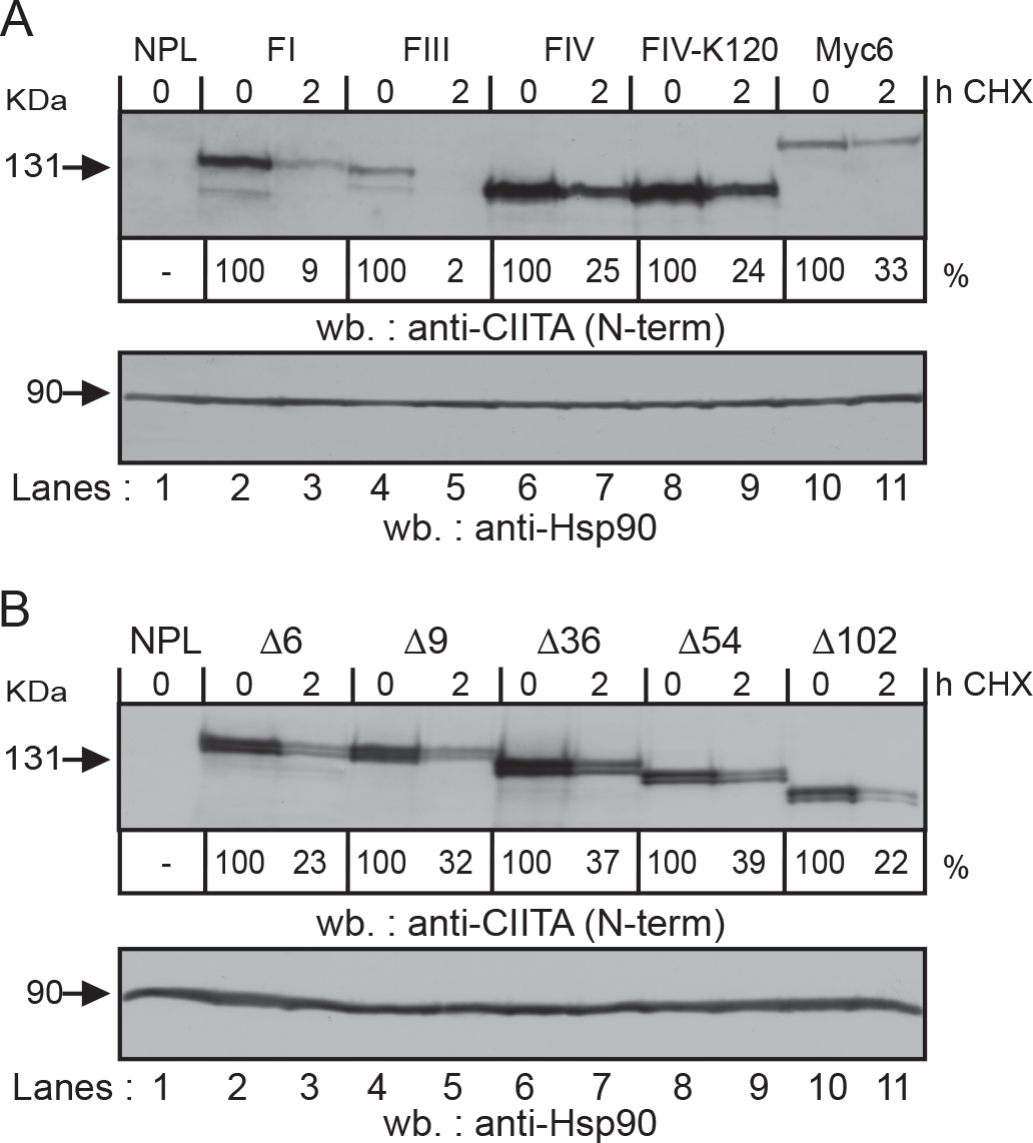
Determination of protein half-life of different CIITA isoforms and mutants by western blotting after cycloheximide treatment. **A, B)** HEK293-EBNA cells stably transfected with the indicated isoforms and deletion mutants of CIITA were either left untreated (0 h), or cultured for 2 h in the presence of 200 μg/ml CHX before harvesting. CIITA protein expression was analyzed by western blotting with CIITA-specific antiserum K5. Protein expression levels were determined by densitometry analysis of x-ray films and are shown below each lane. Blots were stripped and re-probed with a Hsp90-specific antiserum as loading control.

To determine whether increased steady-state levels of expression were due to increased protein stability, we analyzed CIITA turnover by western blotting after cycloheximide (CHX) treatment. We obtained very similar results for CIITA-FIII, -∆36, -∆54, and -∆102 with this approach compared to our earlier pulse chase experiments (Fig 3) [24]. CIITA-FIII was the form with the shortest half-life of approximately 30 min (Fig 3A, lanes 4, 5; S5A Fig). The N-terminal end of CIITA-FIII has to remain free, since addition of an N-terminal Myc_6_-epitope tag stabilizes the protein as we show here by CHX treatment (Fig 3A, lanes 10, 11) and as we showed earlier in pulse chase experiments [24]. The rapid turnover of CIITA-FIII in transfected cells reflects the physiological situation, since we found a similar turnover rate also for endogenous CIITA-FIII in Raji cells in pulse chase experiments (S5B Fig). At the steady-state, CIITA-FI was generally expressed at higher levels compared to CIITA-FIII (Fig 2C, lanes 2, 3; Fig 3A, lanes 2, 4; S5C Fig). Western blotting after CHX treatment suggested that CIITA-FI was more stable than CIITA-FIII (Fig 3A, lanes 2–5). We obtained similar results in transient transfections in HeLa cells by western blotting after CHX treatment (S5C Fig). Thus, the difference in turnover between CIITA-FI and -FIII is possibly sufficient to explain the difference in expression levels.

Compared to CIITA-FIII, all other forms tested, including both forms of CIITA-FIV (K_120_ and I_120_E_121_), -∆6 and -∆9, showed increased stability (Fig 3A and 3B). For these and similar experiments shown below, we used a CIITA-specific antiserum raised against amino acids 25–408 of CIITA (serum K5, [30]. Serum K5 shows a better sensitivity than serum K22 on full length CIITA, allowing more precise quantification (see also Materials and Methods).

Thus the integrity of the extreme N-terminal end of CIITA-FIII is necessary for rapid turnover and transactivation.

### The first ten amino acids of CIITA-FIII behave like a portable degron and transactivation domain

**Fig 4:**
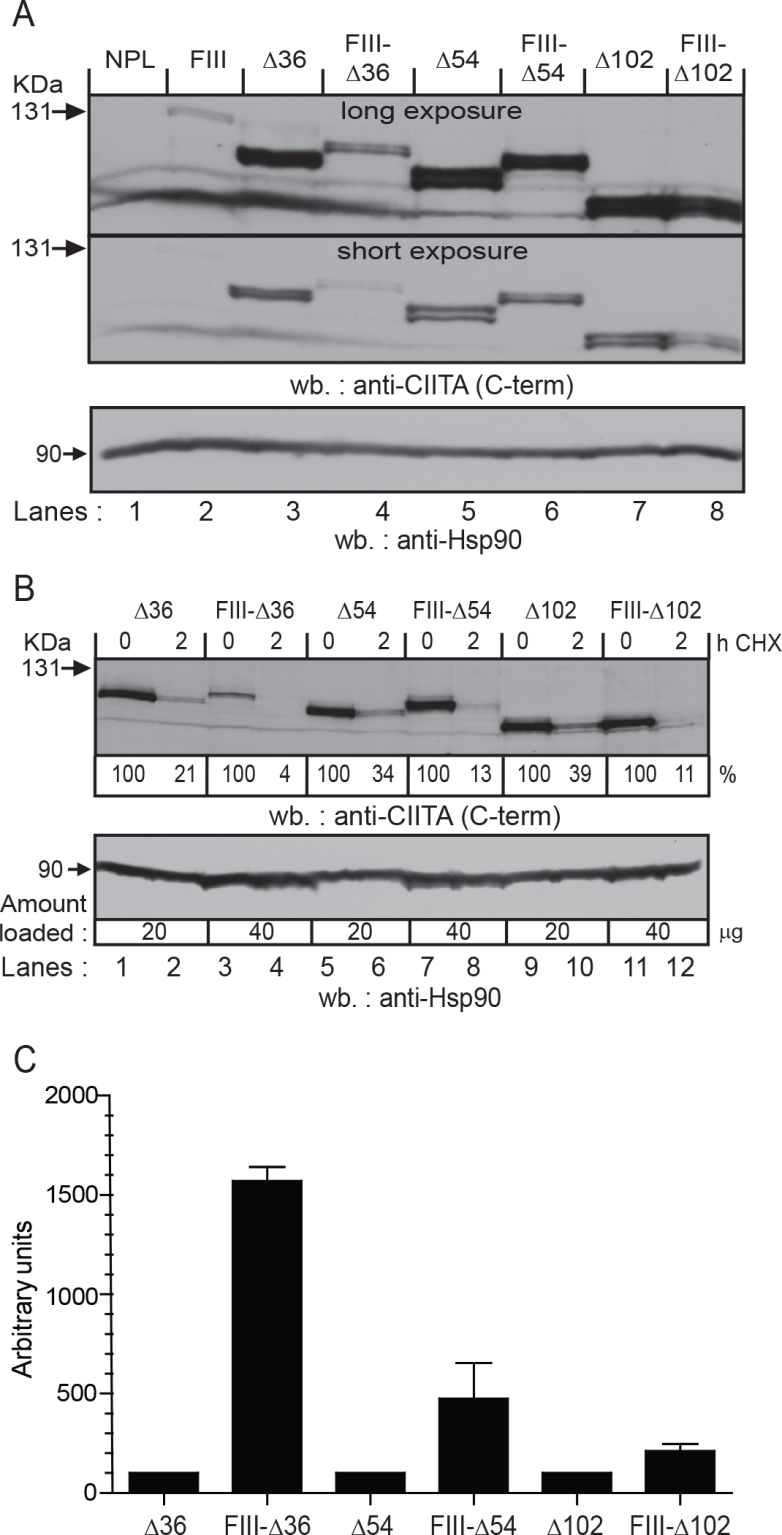
The first ten amino acids of CIITA-FIII act like a portable N-terminal degron and activation domain. **A)** Western blot analysis for CIITA protein expression of the indicated stable transfectants in HEK293-EBNA cells. CIITA was detected with serum K22. A long and a short exposure of the same western blot are shown. **B)** Protein turnover of the different CIITA forms was determined as in Fig 3. For this blot, 40 μg of protein was loaded for the constructs containing the added FIII ends (lanes 3, 4, 7, 8, 11, 12) and 20 μg for those without (lanes 1, 2, 5, 6, 9, 10). **C)** The relative transactivation potential of different CIITA forms was determined as in Fig 2D. The activities of ∆36, ∆54, and ∆102 were set at 100. Values are derived from three experiments.

Since even very short deletions of the CIITA-FIII led to stabilization of the protein and relative loss of transactivation potential, we wanted to test whether the N-terminal end of CIITA-FIII could act as a portable degradation and transactivation domain. We added the first 10 N-terminal amino acids of CIITA-FIII (MRCLAPRPAG) as an N-terminal extension to CIITA-∆36, -∆54 and -∆102, thereby generating FIII-∆36, FIII-∆54 and FIII-∆102 (Fig 4). The HLA-DR cell surface expression of cells expressing the N-terminally extended forms is highly similar to those with truncated forms (S6B Fig). N-terminally extended forms showed lower steady-state protein expression levels (Fig 4A), which were due to increased turnover (Fig 4B).

**Fig 5:**
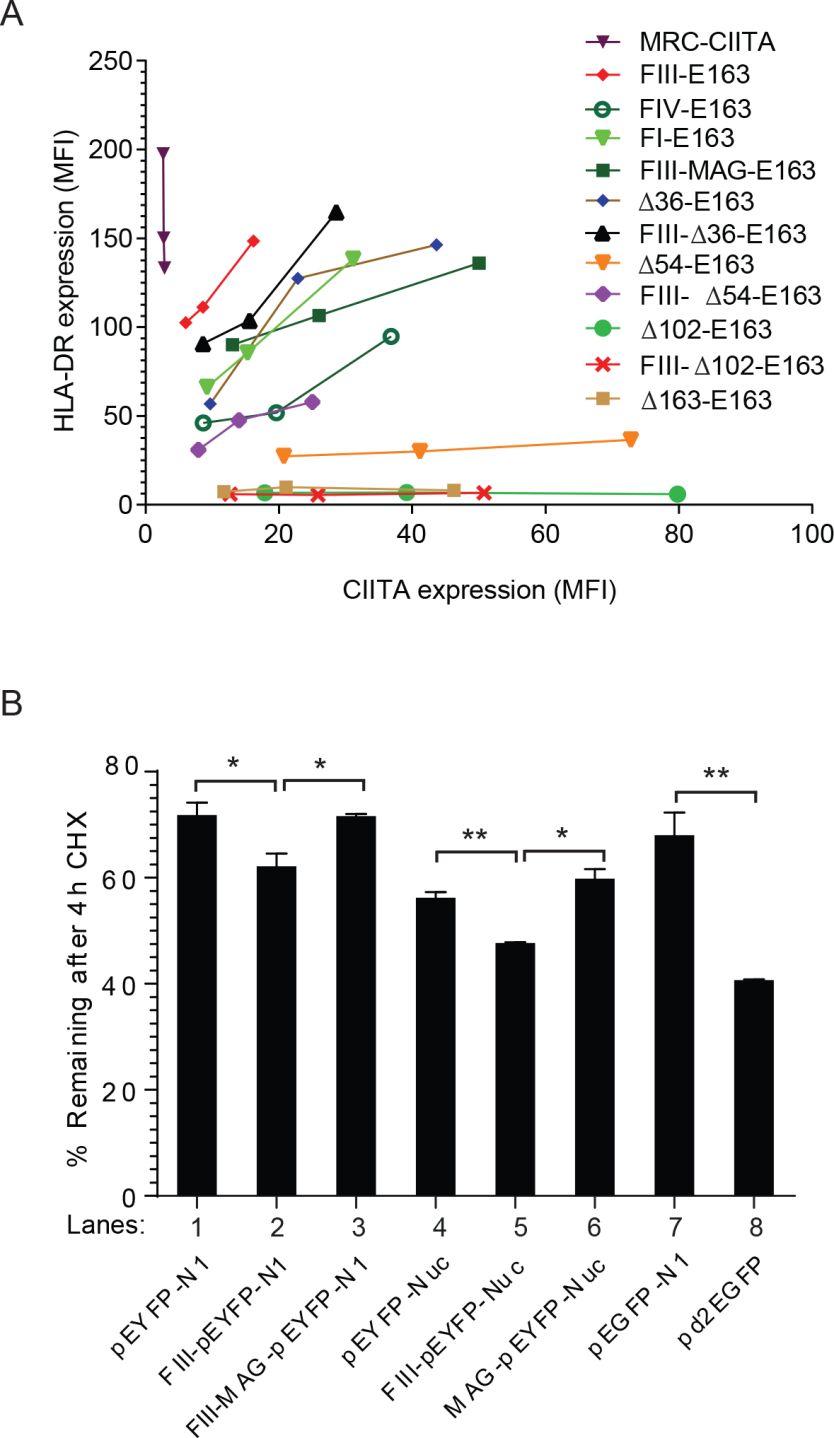
Degradation and transactivation potential of the N-terminal end of CIITA-FIII. **A)** The N-terminal end of CIITA-FIII reduces CIITA protein expression while increasing MHC-II activation. HeLa cells were transiently transfected with the indicated CIITA-E163 constructs and analyzed three days after transfection by flow cytometry for EGFP (FL1) and HLA-DR (FL4) expression as shown for Fig 2E. CIITA MRC is a construct expressing CIITA isoform III without EGFP tag. **B)** CIITA-FIII_1-10_ destabilizes a heterologous protein. pEYFP-N1 (lanes 1–3) or pEYFP-Nuc (3 x SV40-NLS; lanes 4–6) constructs containing N-terminal extensions of the first ten amino acids of CIITA-FIII (FIII-pEYFP, lanes 2, 5), or a CIITA-FIII N-terminal extension with the second and third amino acids (Arg_2_, Cys_3_) replaced by alanine and glycine (FIII-MAG-pEYFP) were transiently transfected into HeLa cells. Shown is the residual EYFP fluorescence after 4h of CHX (40 μg/ml) as determined by flow cytometry. The fluorescence intensity at time point 0h was set as 100%. Lanes 7 and 8 show pEGFP-N1 and pd2EGFP as controls. Experiments were carried out in duplicates. Statistical analysis; student t-test; * p < 0.05; ** p < 0.005.

In order to show more directly the effect of the first 10 amino acids of CIITA-FIII on CIITA expression and MHC-II activation, we made use of the CIITA-E163 constructs (Fig 5A). We transfected different doses of DNA transiently into HeLa cells. Cells were analyzed by flow cytometry for EGFP and HLA-DR expression. As a control without EGFP we used CIITA-FIII-MRC, which codes for a wild-type CIITA-FIII protein, but contains a shortened 5’ UTR with an improved “Kozak” sequence. All other constructs have similar 5’ ends. For all three constructs with an N-terminal extension (FIII-∆36-E163, FIII-∆54-E163, FIII-∆102-E163) we observed a reduction in EGFP expression compared to the non-extended form (∆36-E163, ∆54-E163, ∆102-E163). Concomitantly, we observed an increase in HLA-DR expression for FIII-∆36-E163 and FIII-∆54-E163. The effect is particularly strong for FIII-∆54-E163 (Fig 5A). We also analyzed in this experiment FI-E163, FIV-E163 and FIII-MAG-E163 (see Fig 2E), which all showed higher EGFP and lower HLA-DR expression compared to FIII-E163. These experiments indicate that N-terminal addition of the first 10 amino acids of CIITA-FIII confers increased turnover and a higher transactivation potential to the stabilized truncated forms of CIITA (Figs 4C and 5A).

In order to determine whether the first 10 amino acids of CIITA-FIII were able to destabilize a heterologous protein, we generated N-terminal fusions of CIITA-FIII_1-10_ and EYFP (Fig 5B). Since NTU has been proposed to function mainly in the nucleus, we compared constructs with and without C-terminal NLS (pEYFP-Nuc). As controls, we used untagged EYFP and constructs with an N-terminal extension of CIITA-FIII-MAG_1-10_. Since EYFP is a very stable protein, we treated the transiently transfected HeLa cells with CHX for 2 and 4 h and measured green fluorescence (FL1) by flow cytometry. As a control for degradation, we compared the turnover of EGFP with that of d2EGFP, which contains a strong C-terminal degron derived from mouse ornithine decarboxylase (MODC) [40]. Fig 5B shows the level of residual fluorescence after 4 h of CHX treatment for the different constructs. N-terminal addition of CIITA-FIII_1-10_ led to a significant destabilization of both EYFP and EYFP-Nuc. Residual protein expression was lower in all three C-terminal NLS containing constructs, indicating that CIITA-FIII_1-10_ works similarly, whether it is exclusively expressed in the nucleus or distributed throughout the cell. Subcellular localization of all constructs was as expected, with C-terminally untagged constructs expressed uniformly throughout the cytoplasm and nucleus, and the constructs with a C-terminal NLS showing an exclusively nuclear localization, as detected by fluorescence microscopy (data not shown). Interestingly, the destabilizing effect of CIITA-FIII_1-10_ was completely lost when amino acid positions 2 and 3 (Arg_2_, Cys_3_) were exchanged for alanine and glycine (FIII-MAG-pEYFP and FIII-MAG-pEYFP-Nuc; Fig 5B, lanes 3, 6). These results indicate that the N-terminal end of CIITA-FIII can destabilize also a heterologous protein and that this effect is dependent on the integrity of the extreme N-terminal end.

### N-terminal ends of other NTU proteins can act as portable degrons and transactivation domains

**Fig 6:**
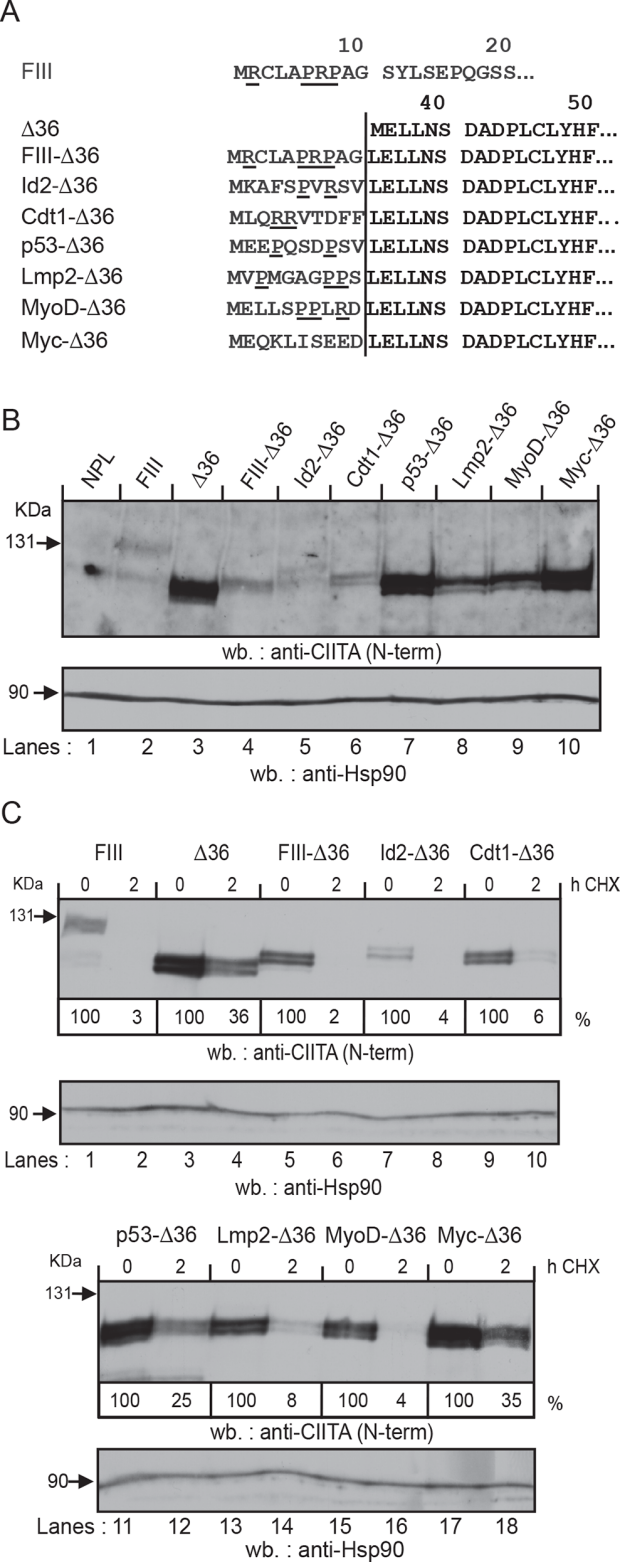
N-terminal ends of NTU proteins increase turnover and transactivation of ∆36. **A)** N-terminal end sequences of the different constructs used. Arginine (R) and proline (P) residues in the added N-terminal ends are underlined. **B)** Western blot analysis for CIITA protein expression of stable transfectants in HEK293-EBNA of the indicated constructs. CIITA was detected with serum K5. **C)** Protein turnover of the different CIITA forms was determined as in Fig 3.

We next added the first 10 amino acids of the previously characterized NTU substrates Id2, Cdt1, Lmp2, and MyoD [12] as N-terminal extensions to CIITA-∆36 (Fig 6A). We tested also the first 10 amino acids of p53, which contains an N-terminal AAD, but has not been analyzed as a substrate for the NTU pathway. As a negative control we added the myc epitope tag, which has been shown to stabilize several NTU proteins and also CIITA-FIII, when added as a six fold repeat at the N-terminus of these proteins [12, 24] (Fig 3A, lanes, 10, 11). Highest steady-state protein expression levels were found for ∆36, p53-∆36 and Myc-∆36 (Fig 6B, lanes, 3, 7, 10), intermediate levels for Lmp2-∆36 and MyoD-∆36 (lanes 8, 9) and lowest levels for CIITA-FIII, FIII-∆36, Id2-∆36 and Cdt1-∆36 (lanes 2, 4, 5, 6). All N-terminal extensions of known NTU substrates increased the turnover of ∆36 (Fig 6C). As expected, N-terminal addition of the myc-tag had no effect on turnover of ∆36, while the first 10 amino acids of p53 induced a minor destabilization (Fig 6C, lanes 11, 12, 17, 18). Thus the N-terminal ends of these NTU proteins also can confer rapid turnover to a heterologous protein such as CIITA.

All constructs tested activated HLA-DR cell surface expression as determined by flow cytometry (S6C Fig). The N-terminal ends of Id2, Cdt1 and MyoD all increased the transactivation potential of ∆36, with Id2 showing the strongest effect. N-terminal addition of the first 10 amino acids of p53, Lmp2 or the myc-tag did not increase the transactivation potential of ∆36, even though Lmp2 increased the turnover of the construct.

### Analysis of amino acid residues contributing to degradation

**Fig 7:**
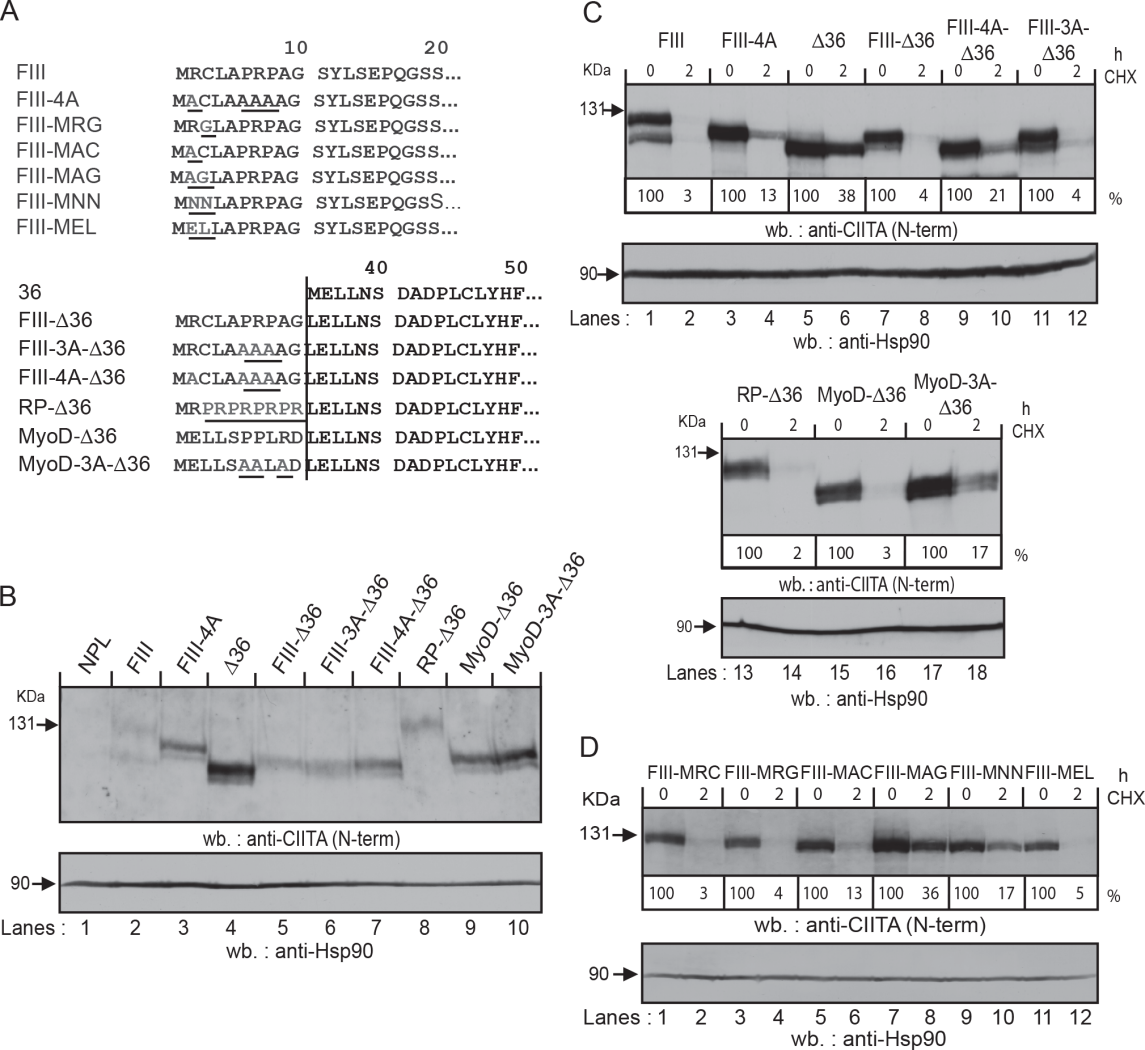
Amino acid analysis of N-terminal degradation sequences. **A)** N-terminal end sequences of mutated constructs based on full length CIITA-FIII (upper) or ∆36 with N-terminal extensions (lower). Mutated amino acid residues are underlined. **B)** Western blot analysis for CIITA protein expression of stable transfectants of the indicated constructs in HEK293-EBNA cells. CIITA was detected with serum K5. **C, D)** Protein turnover of the different CIITA forms was determined as in Fig 3.

No consensus sequence has been identified so far for NTU substrates [12]. However, when we compared the sequences we found that they all contain several arginine and/or proline residues within their first 10 amino acids (Fig 6A). We therefore replaced these residues with alanine residues either in CIITA-FIII or in ∆36 constructs with N-terminal extensions of the first 10 residues of CIITA-FIII or MyoD (Fig 7). Replacement of Arg_2_, Pro_6_, Arg_7_ and Pro_8_ in CIITA-FIII (FIII-4A; Fig 7B, lane 3; Fig 7C, lanes 3, 4) or FIII-∆36 mutant (FIII-4A-∆36, Fig 7B, lane 7; Fig 7C, lanes 9, 10) both led to an increase in stability. However, when only Pro_6_, Arg_7_ and Pro_8_ were exchanged for alanine in the context of an FIII-∆36 mutant, no increase in stability was observed (FIII-3A-∆36, Fig 7B, lane 6; Fig 7C, lanes 11, 12), suggesting that arginine in position 2 is the most important destabilizing residue in CIITA-FIII_1-10_. On the other hand, replacement of Pro_6_, Pro_7_, and Arg_9_ in MyoD-∆36 with alanine led to an increased stability (MyoD-3A-∆36; Fig 7C, lanes 17, 18). We also generated a mutant where residues 2–10 consisted only of alternating arginines and prolines (RP-∆36; Fig 7C, lanes 13, 14). This form was turned over at least as rapidly as CIITA-FIII. Taken together, these results show that arginine and proline residues can contribute to N-terminal turnover.

In order to further define the transcriptional activation and destabilizing element at the N-terminal end of CIITA-FIII, we introduced point mutations in amino acid positions two and three (Arg_2_Cys_3_) of CIITA-FIII (Fig 7D). Stable transfectants of these forms in HEK293-EBNA cells all showed similar levels of HLA-DR expression (S6E Fig). CIITA-FIII-MRC shows the same turnover rate as CIITA-FIII (Fig 7D, lanes 1, 2). A single amino acid exchange at position two (FIII-MAC) induced a partial stabilization (lanes 5, 6), while substitution of cysteine at position three alone (FIII-MRG, lanes 3, 4) did not have any stabilizing effect. However, simultaneous exchange of both amino acids led to a stabilization, which is comparable to CIITA-∆36 (FIII-MAG, Fig 7D, lanes 7, 8). We also tested the potential contribution of amino acid residues at positions two and three from CIITA-FI (N_2_N_3_) and CIITA-FIV (E_2_L_3_). CIITA-FIII-MNN showed an increased level of residual protein expression after CHX treatment, suggesting that replacement of arginine and cysteine with asparagine at positions two and three can have a partially stabilizing effect, at least when analyzed in the context of CIITA-FIII (Fig 7D, lanes 9, 10). On the other hand CIITA-FIII-MEL (Fig 7D, lanes 11, 12) showed a turnover rate comparable to wild-type CIITA-FIII. Altogether these results show that arginine and proline residues close to the N-terminal end contribute to rapid turnover. Arginine in position 2 in CIITA-FIII appears to be important for turnover, however the results also indicate that amino acid residues other than arginine at position 2 are compatible with rapid turnover and that residues further downstream can influence the turnover rate.

### CIITA-FIII binds efficiently to the HLA-DRA promoter *in vivo*

The previous experiments showed that the N-terminal ends of CIITA-FIII and of other NTU proteins could serve as combined degradation signals and transactivation domains. In order to obtain indications how turnover is linked to transactivation potential, we analyzed *in vivo* binding of different isoforms and mutants of CIITA to the HLA-DRA promoter via chromatin immunoprecipitation (ChIP) assay. *In vivo* promoter recruitment of all forms of CIITA tested was similar, including CIITA-Δ54 and -Δ102 (S7B Fig). Taking into account the lower steady-state protein expression levels of CIITA-FIII compared to the other forms (see Fig 2) and the fact that HLA-DR expression is not at saturation levels in these cells (S6A Fig), this experiment suggests that CIITA-FIII is recruited more efficiently to the HLA-DRA promoter than the other more highly expressed forms. In addition, the ChIP experiment also shows that the observed reduced transactivation potential of the CIITA-∆54 and -∆102 forms is not due to reduced *in vivo* recruitment to the HLA-DRA promoter.

In order to compare the rate of transcription initiation between the different CIITA forms, we determined the levels of unspliced (nascent) HLA-DRA transcripts in the different transfectants using primers in exon 1 and intron 1 of the HLA-DRA gene (S7A Fig). Nascent HLA-DRA transcript levels were highly similar to spliced mRNA levels (S7C Fig) suggesting that the observed differences in transcriptional activities of the different CIITA forms are not due to differences in transcriptional initiation versus elongation.

### CIITA-FIII interacts more efficiently with components of the transcription machinery and MHC-II promoter binding proteins

**Fig 8:**
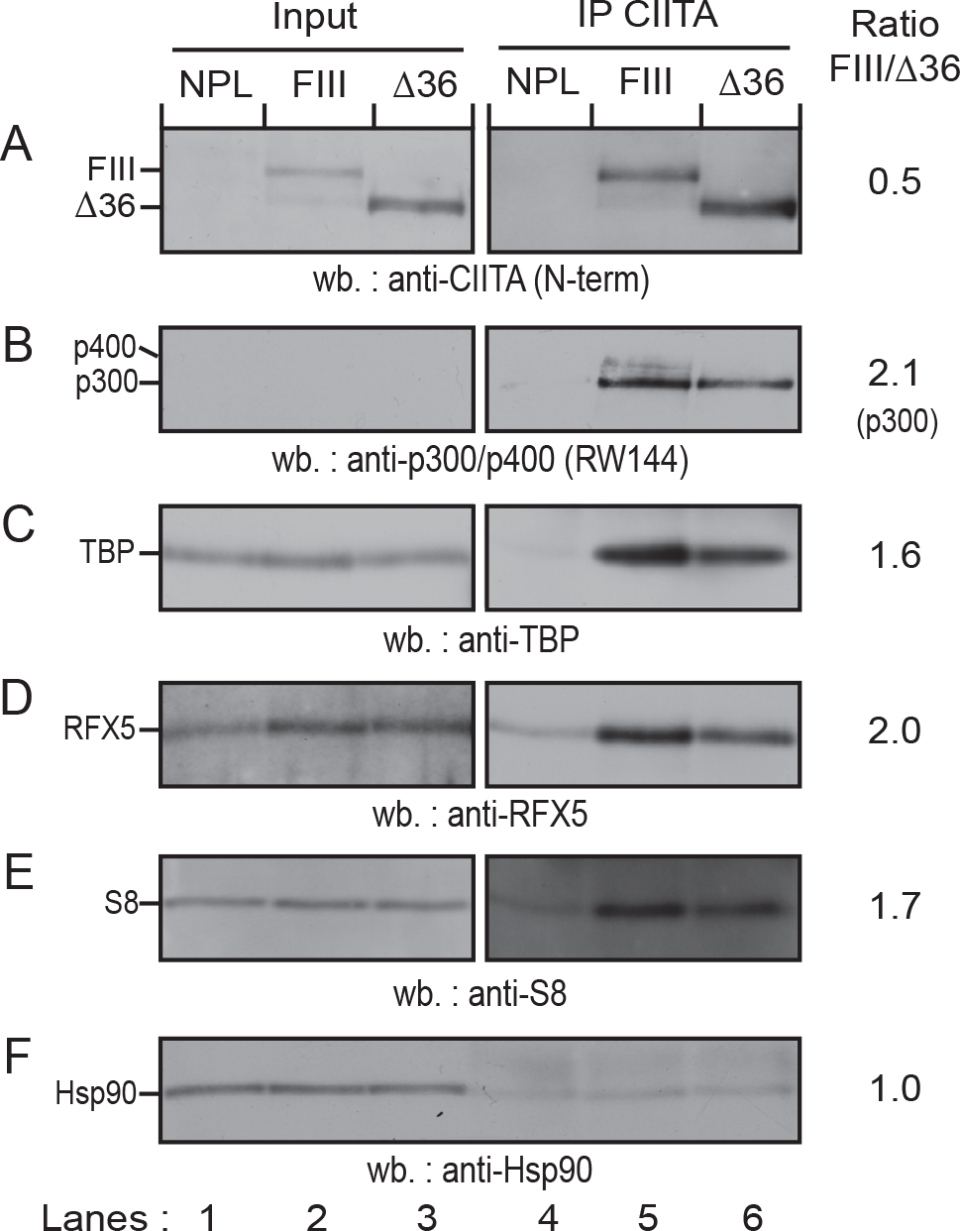
CIITA-FIII interacts more efficiently with protein partners. CIITA was immunoprecipitated from protein extracts of HEK293-EBNA cells stably transfected with empty EBS-NPL vector (lanes 1, 4), CIITA-FIII (lanes, 2, 5), or CIITA-∆36 (lanes 3, 6) respectively. Input controls (lanes 1–3) or immunoprecipitated material (lanes 4–6) were separated by SDS-PAGE (8% gel), blotted and analyzed by western blotting. The membrane was cut in half and the upper part was probed with antibodies for CIITA **(A)**, stripped, and reprobed consecutively with antibodies for p300/p400 (antibody RW144) **(B)**, RFX **(D)**, and Hsp90 as a control **(F)**, the lower part was hybridized with antibodies against TBP **(C)**, stripped and reprobed for S8 using Reliablot secondary reagents **(E)**. For input controls longer exposures are shown, with the exception of Hsp90. Ratios of band intensities of bands in lane 5 versus lane 6 are shown on the right.

TADs work primarily by recruiting components of the transcription machinery to promoters [41]. Increased recruitment of CIITA-FIII might therefore be due to more efficient interactions with such proteins. We therefore immunoprecipitated CIITA-FIII or the stabilized mutant CIITA-∆36 from stable transfectants and tested for the presence of co-immunoprecipitated proteins by western blotting (Fig 8). About twofold more CIITA protein was immunoprecipitated from cells expressing CIITA-∆36 compared to CIITA-FIII (Fig 8A). Despite this fact, both the coactivator p300 and the TATA-box binding protein (TBP) were co-immunoprecipitated more efficiently with CIITA-FIII (Fig 8B and 8C). The RW144 mAb recognizes both p300 and the chromatin remodeling protein p400/Domino [32], and we observed a high molecular weight band corresponding to p400 only in the co-immunoprecipitate of CIITA-FIII (Fig 8B, lane 5). CIITA-FIII also interacted more efficiently with the RFX5 subunit of the RFX complex (Fig 8D), which binds to the MHC-II promoter X-box element [42]. Previously, Greer and colleagues showed that the 19S proteasome ATPase S8 (Sug1) co-immunoprecipitates with CIITA and that knockdown of S8 led to reduced *in vivo* recruitment of CIITA to the HLA-DRA promoter in IFN-γ treated HeLa cells [43]. We found that S8 also was co-immunoprecipitated more efficiently in cells expressing CIITA-FIII (Fig 8E). Taking into account the twofold lower amount of immunoprecipitated CIITA-FIII (Fig 8A), this experiment indicates a three- to fourfold more efficient interaction between CIITA-FIII and its protein partners compared to CIITA-∆36.

### Effect of p300/CBP knock out on CIITA expression

**Fig 9:**
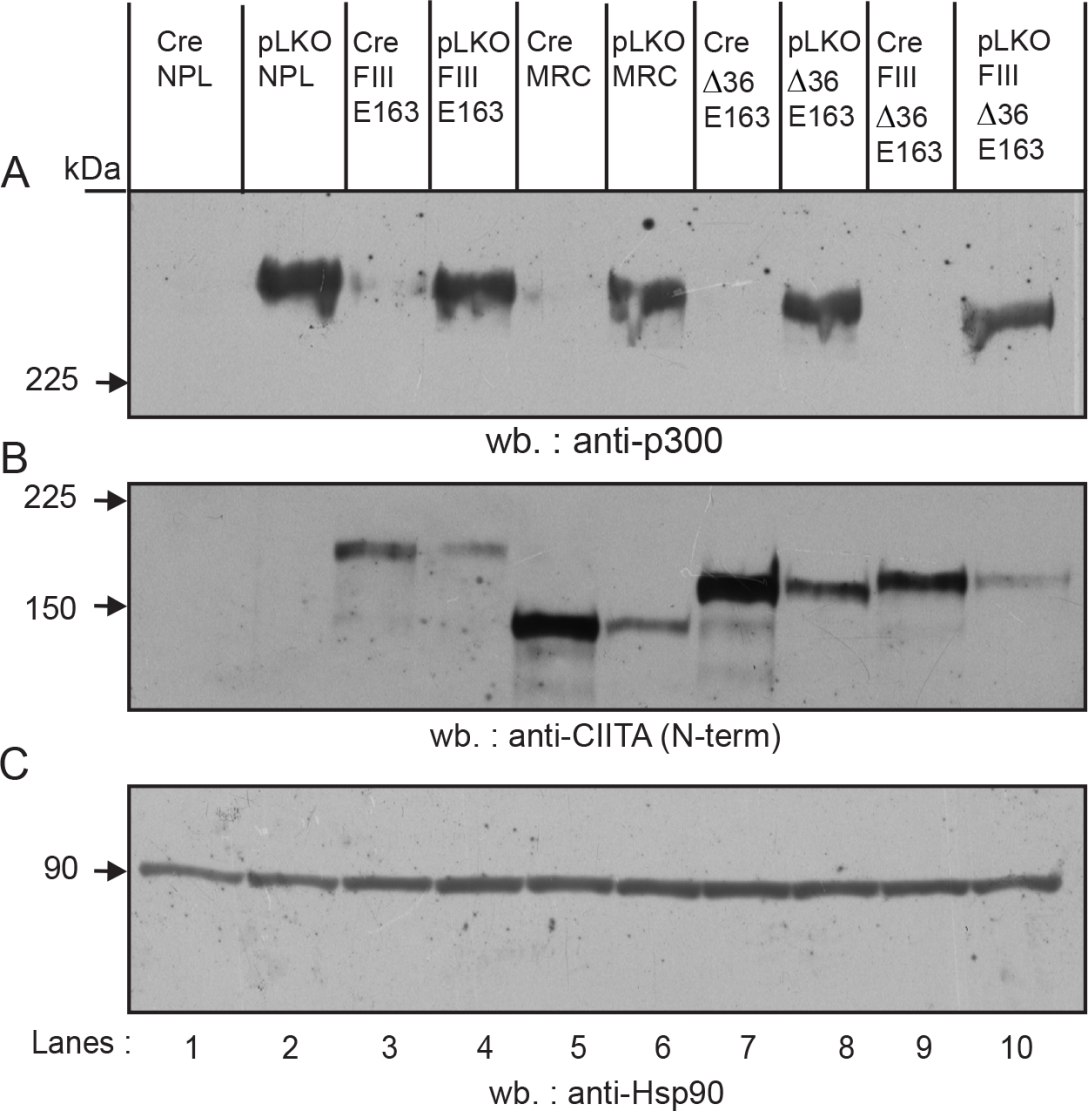
Knock out of p300/CBP affects expression of CIITA independently from the FIII N-terminal end. MEF-p300^fl/fl^-CBP^fl/fl^ cells were infected either with a Cre-recombinase expressing lentivirus (Cre; lanes 1, 3, 5, 7, 7, 9) or with empty lentivirus vector (pLKO; lanes 2, 4, 6, 8, 10). One day after infection, the cells were transiently transfected with EBS-NPL empty vector (lanes 1, 2), CIITA-FIII-E163 (lanes 3, 4), CIITA-FIII-MRC (lanes 5, 6), CIITA-∆36-E163 (lanes 7, 8), and CIITA-FIII-∆36-E163 and analyzed three days after transfection. Steady state protein expression levels were analyzed by SDS-PAGE followed by western blotting. The blots are from a single membrane, which was cut into horizontal strips, and show immunoblotting with antibodies against p300 (A), CIITA (B), and Hsp90 (C).

The more efficient interaction between CIITA-FIII and the coactivator p300 was intriguing, since p300 has been shown to act as an E4 ubiquitin ligase for p53 [44]. In order to analyze a possible role of p300 and its homolog CBP in the activity of the CIIITA FIII N-terminal end, we made use of a mouse embryonal fibroblast (MEF) cell line containing floxed alleles for both p300 and CBP [26]. Cre recombinase was expressed in these cells via infection with a Cre-expressing lentiviral vector. Control cells were infected with empty lentivirus vector. Efficient Cre-mediated deletion of p300/CBP was confirmed by western blotting for p300 (Fig 9A). One day after infection, the cells were transiently transfected with various CIITA-E163 constructs and analyzed 3 days later by western blotting (Fig 9). These experiments showed that absence of p300/CBP leads to an increase in CIITA expression, however this effect was not only observed for CIITA FIII, but also for CIITA ∆36 (Fig 9). This suggests that the effect of p300/CBP is not mediated through the FIII N-terminal end. Rather, these results are in agreement with a report that mapped a CBP interaction domain to region 59 to 94 in CIITA-FIII [45].

## Discussion

We show here that the extreme N-terminal end of CIITA isoform III acts as a portable degron and transactivation element. Several lines of evidence suggest that this degradation is mediated through the NTU pathway. In the majority of proteins the N-termini are processed in several different ways and only a minority of proteins have a free α-amino group at the N-terminus. Only proteins containing amino acids in position 2 with large side chains are resistant to N-terminal modification [13, 46]. Arginine is the amino acid with the bulkiest side chain, and thus CIITA-FIII (MRC…) is predicted to possess a free methionine group at its N-terminus. Addition of an N-terminal Myc-tag stabilizes CIITA-FIII, as has been shown for other NTU substrates [11, 24, 47]. Evidence for the NTU pathway has been mostly indirect, and only few interactions of ubiquitin with an N-terminal α-amino group have so far been demonstrated, mostly *in vitro* [48–52].

Addition of the first 10 amino acids of CIITA-FIII increases not only the turnover of stabilized truncated CIITA proteins, but destabilizes also a very stable heterologous protein, such as EYFP. In both cases the integrity of the extreme N-terminal end of CIITA-FIII_1-10_ (positions 2 and 3) is essential for the destabilizing function. Thus the N-terminal end of CIITA-FIII behaves like a portable degron. Interestingly, the N-terminal ends of several known NTU substrates were also able to induce increased turnover on CIITA-∆36. No consensus sequence for the NTU pathway has been defined [12], but we found that all sequences contained several proline and arginine residues. Mutational analysis indicated that these residues contribute to rapid degradation and an artificial N-terminal end composed only of proline and arginine residues was also able to destabilize CIITA-∆36.

While the present work provides a first analysis of the contribution of specific residues to N-terminal turnover, the rules for the recognition of degradation substrates need to be clarified further. The situation is particularly intriguing for glutamate in the second position, which is the smallest of the amino acids that may be resistant to cleavage by methionine amino peptidase [46]. Several known NTU substrates tested here and by other groups contain glutamate in position 2, such as MyoD (MELL…), LMP1 (MERD…), and p16INK4a (MEPA…) [11, 47, 48]. On the other hand, several N-terminal ends of stabilized proteins tested here also contain glutamate at position 2: CIITA-FIV (MELG…), CIITA-∆36 (MELL…), and the myc-tag (MEQK…; see Fig 6). Furthermore, when we replaced amino acid positions 2 and 3 in CIITA-FIII with those of CIITA-FIV, this construct was still rapidly turned over (FIII-MEL; Fig 7D). Thus N-terminal ends of proteins with glutamate at position 2 may be stabilizing or destabilizing, most probably depending on residues further downstream. Arginines and prolines probably play an important role in this context.

We show here for the first time that the extreme N-terminal end of a protein not only induces protein turnover, but contributes also to transcriptional activation, and that this activity can be transferred via very short sequences to a heterologous protein. It is essential that the N-terminal end of this sequence remains free, since N-terminal epitope tagging leads to stabilization and relative loss of transactivation potential (Fig 2E, S3 Fig) [24]. While the contribution of the N-terminal end of CIITA-FIII to transcriptional activation is quite evident, it should be pointed out that this is a relative contribution to transcriptional activation, and CIITA forms that have higher stability and a resulting higher steady-state level of expression such as CIITA-FIV and CIITA-∆36 show a similar overall activity of MHC-II activation. Almost invariably, TADs contain multiple, sometimes very short sequence elements that appear to work cooperatively, and which show little activity when expressed on their own [53]. CIITA is no exception and our N-terminal truncation and mutation analysis indicates that, in addition to the N-terminal end of CIITA-FIII, at least three regions within the N-terminal AAD (regions 36–54, 54–102, and downstream of position 102) contribute to transactivation (this work, and [24]). Fontes and colleagues showed that internal deletions of residues 51–71 and 84–103 reduced transcriptional activity of CIITA [54] and residues 59–94 have been shown to interact with the co-activator CBP [45]. Our N-terminal deletion analysis indicates that residues 36–54 make a major contribution to the transactivation potential of CIITA.

All sequence elements of CIITA with transcriptional activity also contribute to turnover. This is not only true for the N-terminal end of CIITA-FIII but also for the internal regions within the CIITA AAD contributing to transactivation. While the N-terminally truncated forms of CIITA are stabilized when compared to CIITA-FIII, they remain proteins with a relatively rapid turnover and CIITA-FIV, -∆36, -∆54 and -∆102 all show half-lives of less than 2 hours. Thus, we have been unable to dissociate turnover from transactivation. In agreement with our results, Bhat and colleagues found that expression of an HLA-DRA dependent reporter gene was reduced, when CIITA-transfected and IFN-γ induced HeLa cells were incubated with the proteasomal inhibitor MG132 [43]. On the other hand, Greer and colleagues had reported earlier that mono-ubiquitination of CIITA contributed to transactivation in a non-degradative manner [25]. This conclusion was mainly based on the finding that overexpression of a ubiquitin mutant, in which all lysines were mutated to arginine (“mono-ubiquitin”), increased the Flag-CIITA dependent expression of an HLA-DRA promoter reporter gene and recruitment to the HLA-DRA promoter in ChIP assays [25]. CIITA protein expression levels were not analyzed in these experiments. Bhat and colleagues identified three lysines in CIITA (K315, K330, K333), which are mono-ubiquitinated in CIITA and contribute to MHC-II transactivation [39]. Ubiquitination is controlled by serine 280. These experiments were also carried out with stabilized Flag-CIITA. Thus non-degradative mechanisms of CIITA activity can maybe only be uncovered when using stabilized forms of CIITA.

We found here that CIITA-FIII interacts three to fourfold more efficiently with proteins of the transcription machinery, such as p300, p400, and TBP, with MHC-II promoter binding proteins such as RFX5, and with the 19S proteasome ATPase S8 (Sug1). These co-immunoprecipitations most probably reflect interactions in solution, independently of binding to chromatin. While p300/CBP and S8 where shown to interact directly with the AAD of CIITA, the interaction domain of CIITA with RFX5 was mapped to the GTP-binding domain of CIITA [21, 43]. Thus the presence of the N-terminal end of CIITA-FIII mediates more efficient interactions with protein partners also indirectly, possibly in form of a multi-protein complex. This preformed complex may be more efficiently recruited to the MHC-II promoters through multiple interactions. Interaction of CIITA with protein partners in solution would also fit well with our earlier observation that rapid turnover of CIITA is still observed when CIITA-FIII is co-expressed with a strong dominant negative CIITA-mutant lacking the AAD and P/S/T domains (NLS-L335), which competes very efficiently for DNA binding [24, 30, 55]. This suggests that rapid turnover of CIITA-FIII is not dependent on promoter recruitment.

Efficient recruitment of CIITA-FIII and interaction with the 19S ATPase S8 is in agreement with the finding of a direct interaction between CIITA and S8 [56]. Bhat and colleagues mapped the interaction domains to the N-terminal P/S/T domain [56], which we had shown earlier contains one of the destabilizing regions in CIITA (aa 230–260)[24]. Our finding here that the N-terminal end of CIITA-FIII increases co-precipitation of S8, could be due to direct or indirect interactions.

In conclusion, we show here that the N-terminal end of CIITA-FIII is important for rapid protein turnover and transactivation. Increased transactivation potential appears to be mainly due to increased promoter recruitment through more efficient interaction with the transcription machinery and promoter binding proteins. Since several other N-terminal ends of known NTU proteins are able to function very similar to the N-terminal end of CIITA-FIII, it is to be expected that this mechanism also applies to other transcriptional regulators.

## Supporting Information

S1 Fig K_120_ and I_120_E_121_ isoforms of CIITA are generated through alternative splicing. The genomic context of splice junctions of exons 4 and 5 of human CIITA is shown. The splice donor (gt) and alternative splice acceptor (ag) nucleotides are underlined.
(TIF)

S2 Fig CIITA protein quantification through western blotting. **A)** Protein was extracted from HEK293-EBNA cells transiently transfected with 500 ng of CIITA-FIII. The indicated amounts of protein were loaded on a 6% gel, separated and blotted with antiserum K5. **B)** Quantification of bands was carried out as described in Materials and Methods. The quantification shown was obtained from a duplicate of experiments. Note that CIITA protein expression levels in these transient transfections are considerably higher than in stable transfectants (data not shown).
(TIF)

S3 Fig CIITA expression levels and transactivation potential in transient transfections. Transient transfection in HEK293-EBNA cells were carried out as described in S1 Materials and Methods. **A)** Western blot analysis of Myc6-CIITA-FIII (lane 1) and CIITA-FIII (lane 2). CIITA was detected with serum K5. **B)** Western blot analysis of indicated CIITA forms. CIITA was detected with antiserum K22. The figure is derived from a single blot, but intervening bands were cut. **C)** Determination of relative transactivation potential of different CIITA forms based on MFI values of cell surface HLA-DR expression (gated on EGFP-positive cells) compared to CIITA protein expression levels. The activity of CIITA-FIII was set at 100.
(TIF)

S4 Fig Analysis of the phosphorylation of different CIITA forms. Protein extracts from cell lines stably transfected with the indicated CIITA forms were either left untreated (odd lanes numbers) or treated with λ-phosphatase (even lane numbers), separated by SDS-PAGE, blotted and probed with the CIITA-specific antiserum K5.
(TIF)

S5 Fig Protein turnover of transfected CIITA-FI and CIITA-FIII, and of endogenous CIITA in Raji cells. **A)** HEK293-EBNA cells stably transfected with CIITA-FIII were either left untreated (0 h), or cultured for 1, respectively 2 h in the presence of 200 μg/ml CHX before harvesting. CIITA protein expression was analyzed by western blotting with CIITA-specific antiserum K5. Protein expression levels were determined by densitometry analysis of x-ray films and are shown below each lane. **B)** Protein turnover of endogenous CIITA in the Burkitt lymphoma cell line Raji (lanes 2–5) was determined by pulse chase experiment on ^35^S metabolically labeled cells as described [24]. Lane 1 shows an immunoprecipitation reaction at time point 0 h with an extract from RJ2.2.5 [57], a Raji-derived cell line in which both alleles of CIITA are deleted [17] [58]. The position of CIITA is indicated (arrow). Raji cells express predominantly CIITA-FIII [22]. **C)** HeLa cells transiently transfected with CIITA-FIII-MRC (lanes 1–5) or with CIITA-FI (lanes 6–11) were either left untreated (0 min), or incubated for the indicated times with 40 μg/ml CHX before harvesting. SDS-PAGE, western blotting with CIITA-specific antiserum K5, detection and quantification was carried out using a BioRad Chemidoc MP system. The lower image shows western blotting of the same membrane with a Hsp90-specific antiserum as loading control.
(TIF)

S6 Fig HLA-DR cell surface expression of CIITA-transfected cell lines. HLA-DR cell surface expression was determined by staining with the HLA-DR-specific mAB HK14 coupled to Quantum Red (Sigma) and analysis of live cells by flow cytometry (FACScalibur). **A)** Myc-∆36 transfected cells are shown after 1 week (lane 2) or two weeks of hygromycin B selection (lane 3). **B)** HLA-DR expression of the cell lines used for the experiments shown in Fig 4. **C)** HLA-DR expression of the cell lines used for the experiments shown in Fig 5. **D)** HLA-DR expression of the cell lines used for the experiments shown in Fig 6A. **E)** HLA-DR expression of the cell lines used for the experiments shown in Fig 6D (lanes 1–7) and Fig 3B (lanes 8, 9) respectively.
(TIF)

S7 Fig *In vivo* binding to the HLA-DRA promoter and HLA-II gene activation of different forms of CIITA. **A)** Schematic drawing of the HLA-DRA locus and location of primers for gene expression and ChIP analysis. **B)** CIITA/chromatin complexes from the indicated stable transfectants in HEK293-EBNA cells were immunoprecipitated with the CIITA-specific antiserum K22. The HLA-DRA promoter region was amplified using primers F1 and R1 and measured by QPCR. Values and standard errors are from four independent ChIP experiments. **C)** Mature (spliced) and unspliced HLA-DRA mRNA expression was determined by RT-QPCR. Absence of DNA contamination was confirmed by absence of amplification with samples not treated with reverse transcriptase (data not shown). Expression levels of CIITA-FIII expressing cells were arbitrarily set at 100. Values are derived from two of the cell preparations used for the ChIP analysis shown in (B). Statistical analysis revealed that differences of the expression levels between mature and unspliced HLA-DRA RNAs for the different constructs are not significant.
(TIF)

S1 Materials and Methods (DOCX)

S1 Table Q-PCR oligonucleotide primers for gene expression and CHIP analysis. (TIF)
