# Supplementary figures and images for: Degradation, Promoter Recruitment and Transactivation Mediated by the Extreme N-Terminus of MHC Class II Transactivator CIITA Isoform III
Source: PLoS One. 2016 Feb 12;11(2):e0148753. doi: 10.1371/journal.pone.0148753 (PMC4752451; doi:10.1371/journal.pone.0148753)

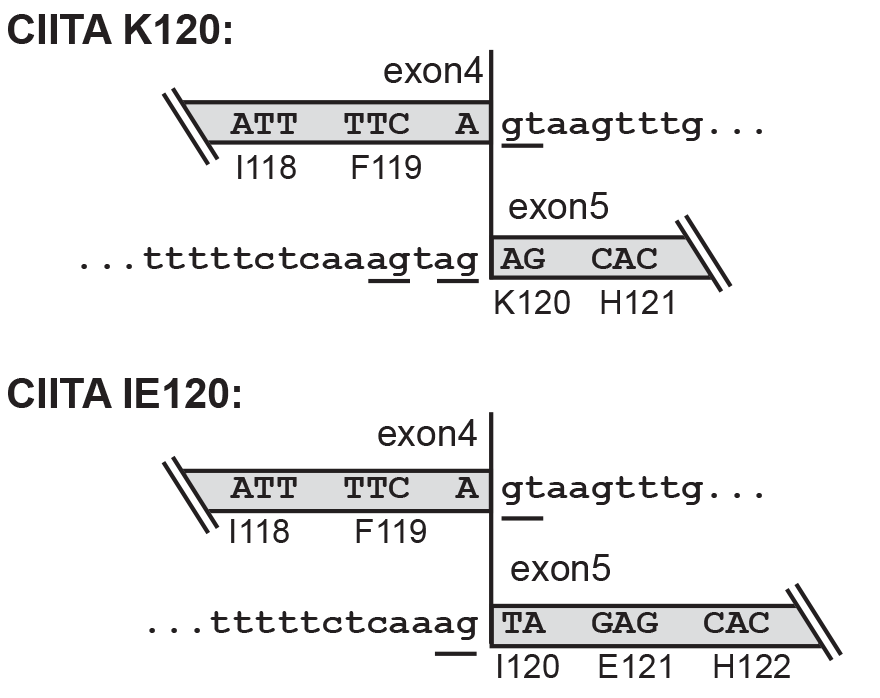

Supplement: S1 Fig — The genomic context of splice junctions of exons 4 and 5 of human CIITA is shown. The splice donor (gt) and alternative splice acceptor (ag) nucleotides are underlined. (TIF) [file pone.0148753.s001.tif]

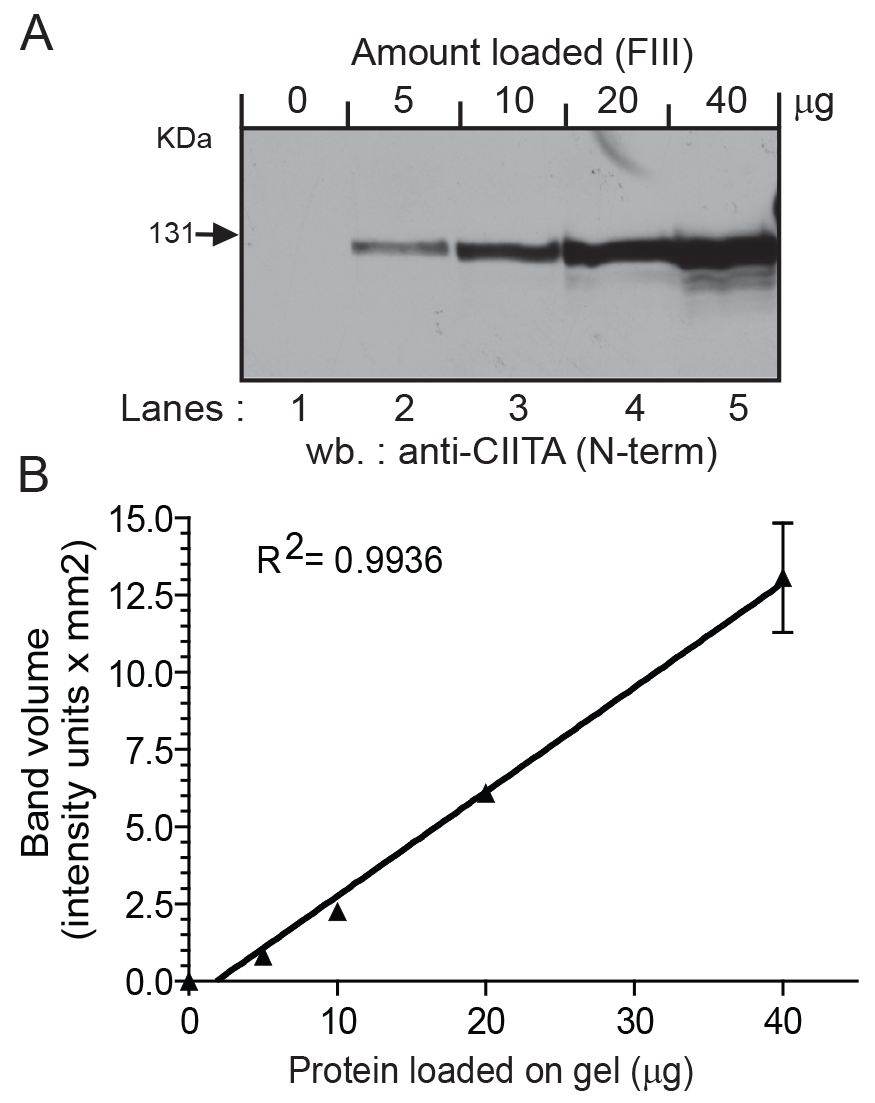

Supplement: S2 Fig — A) Protein was extracted from HEK293-EBNA cells transiently transfected with 500 ng of CIITA-FIII. The indicated amounts of protein were loaded on a 6% gel, separated and blotted with antiserum K5. B) Quantification of bands was carried out as described in Materials and Methods. The quantification shown was obtained from a duplicate of experiments. Note that CIITA protein expression levels in these transient transfections are considerably higher than in stable transfectants (data not shown). (TIF) [file pone.0148753.s002.tif]

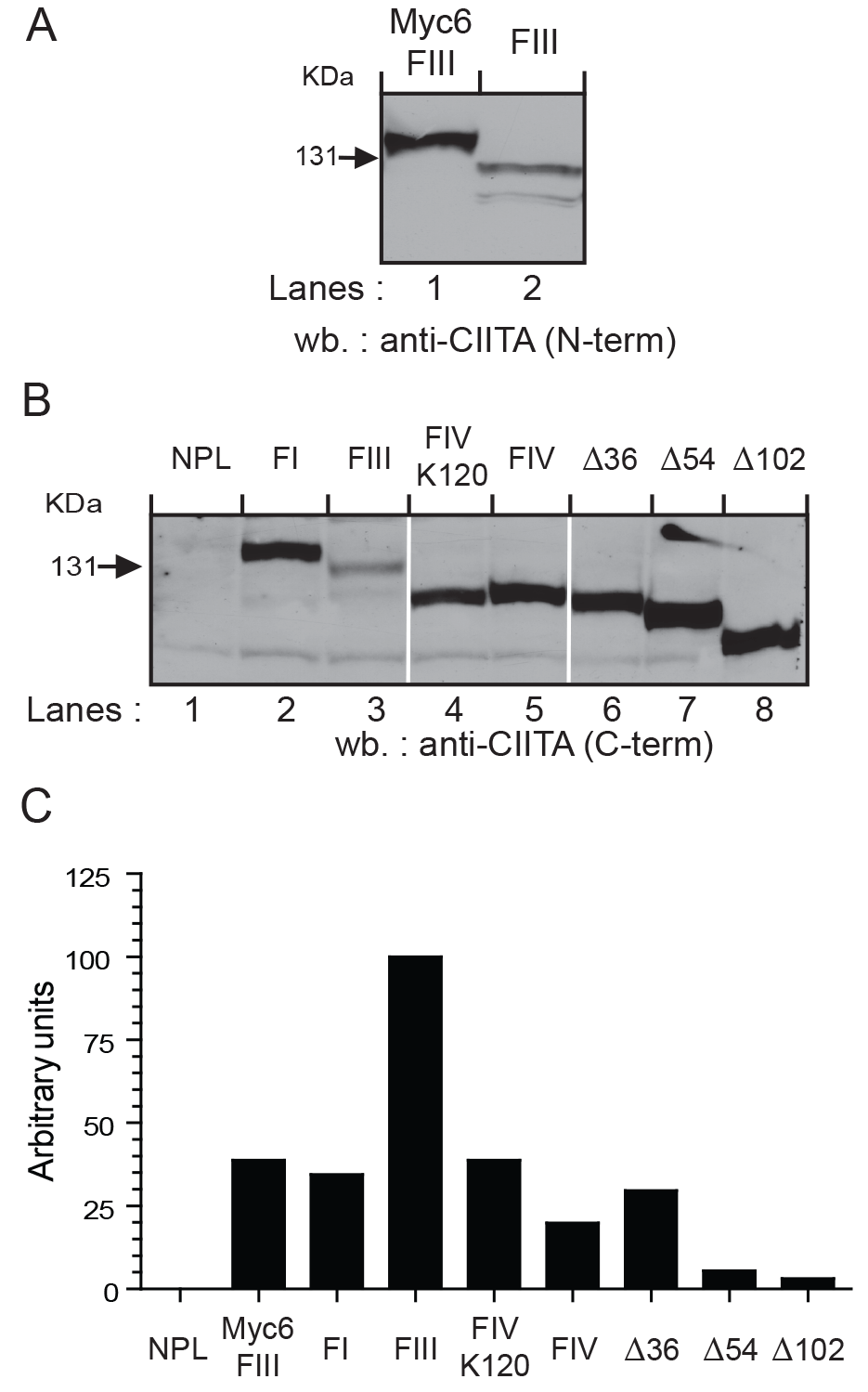

Supplement: S3 Fig — Transient transfection in HEK293-EBNA cells were carried out as described in S1 Materials and Methods. A) Western blot analysis of Myc6-CIITA-FIII (lane 1) and CIITA-FIII (lane 2). CIITA was detected with serum K5. B) Western blot analysis of indicated CIITA forms. CIITA was detected with antiserum K22. The figure is derived from a single blot, but intervening bands were cut. C) Determination of relative transactivation potential of different CIITA forms based on MFI values of cell surface HLA-DR expression (gated on EGFP-positive cells) compared to CIITA protein expression levels. The activity of CIITA-FIII was set at 100. (TIF) [file pone.0148753.s003.tif]

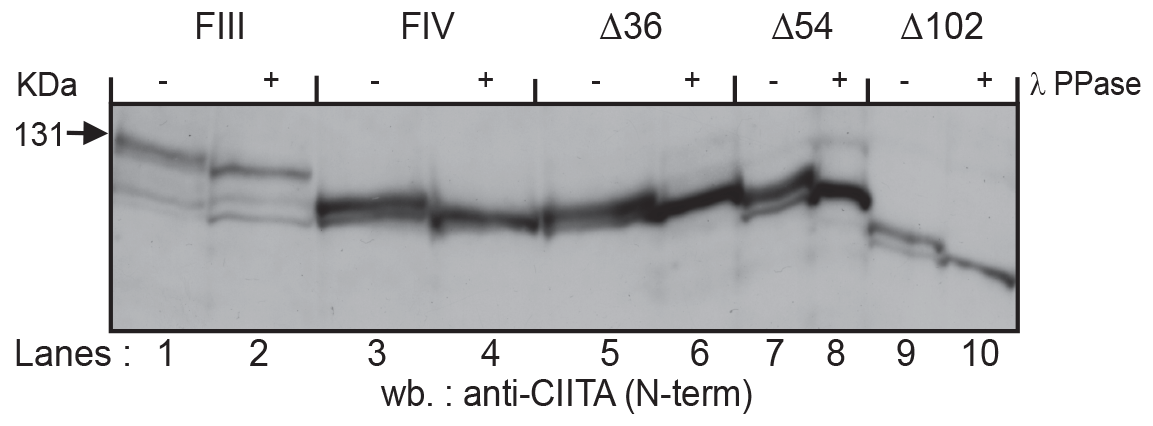

Supplement: S4 Fig — Protein extracts from cell lines stably transfected with the indicated CIITA forms were either left untreated (odd lanes numbers) or treated with λ-phosphatase (even lane numbers), separated by SDS-PAGE, blotted and probed with the CIITA-specific antiserum K5. (TIF) [file pone.0148753.s004.tif]

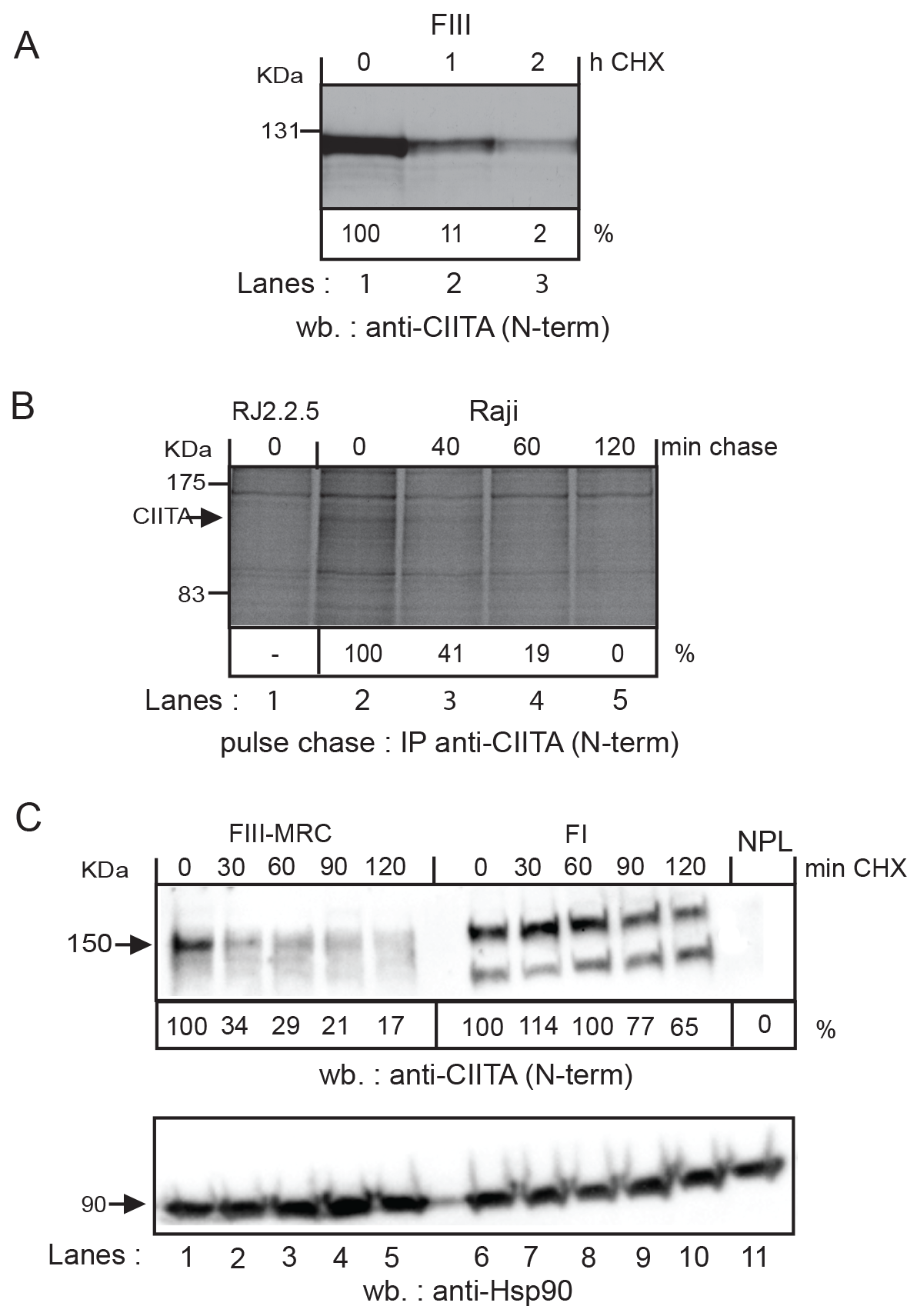

Supplement: S5 Fig — A) HEK293-EBNA cells stably transfected with CIITA-FIII were either left untreated (0 h), or cultured for 1, respectively 2 h in the presence of 200 μg/ml CHX before harvesting. CIITA protein expression was analyzed by western blotting with CIITA-specific antiserum K5. Protein expression levels were determined by densitometry analysis of x-ray films and are shown below each lane. B) Protein turnover of endogenous CIITA in the Burkitt lymphoma cell line Raji (lanes 2–5) was determined by pulse chase experiment on 35S metabolically labeled cells as described [24]. Lane 1 shows an immunoprecipitation reaction at time point 0 h with an extract from RJ2.2.5 [57], a Raji-derived cell line in which both alleles of CIITA are deleted [17] [58]. The position of CIITA is indicated (arrow). Raji cells express predominantly CIITA-FIII [22]. C) HeLa cells transiently transfected with CIITA-FIII-MRC (lanes 1–5) or with CIITA-FI (lanes 6–11) were either left untreated (0 min), or incubated for the indicated times with 40 μg/ml CHX before harvesting. SDS-PAGE, western blotting with CIITA-specific antiserum K5, detection and quantification was carried out using a BioRad Chemidoc MP system. The lower image shows western blotting of the same membrane with a Hsp90-specific antiserum as loading control. (TIF) [file pone.0148753.s005.tif]

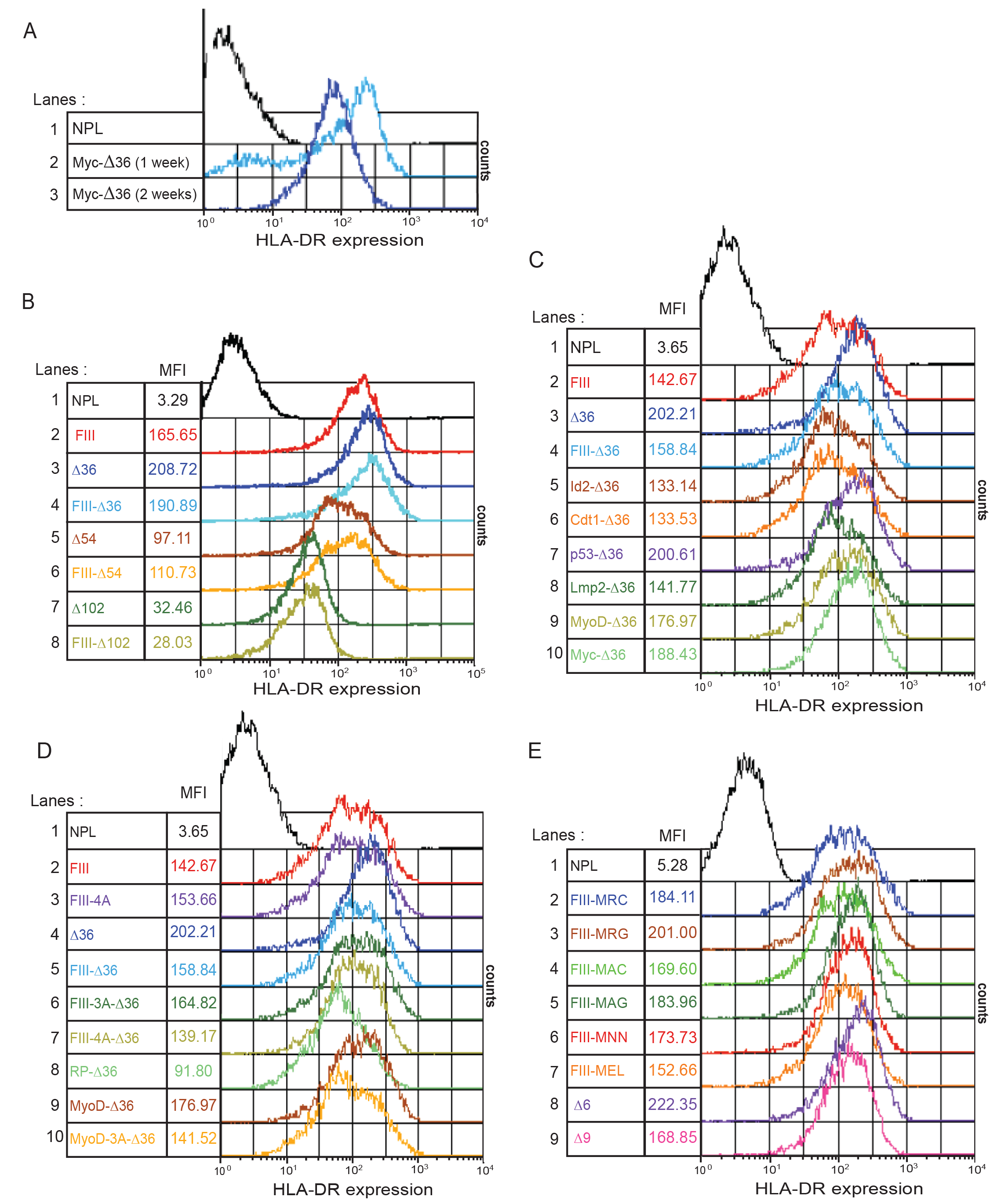

Supplement: S6 Fig — HLA-DR cell surface expression was determined by staining with the HLA-DR-specific mAB HK14 coupled to Quantum Red (Sigma) and analysis of live cells by flow cytometry (FACScalibur). A) Myc-∆36 transfected cells are shown after 1 week (lane 2) or two weeks of hygromycin B selection (lane 3). B) HLA-DR expression of the cell lines used for the experiments shown in Fig 4. C) HLA-DR expression of the cell lines used for the experiments shown in Fig 5. D) HLA-DR expression of the cell lines used for the experiments shown in Fig 6A. E) HLA-DR expression of the cell lines used for the experiments shown in Fig 6D (lanes 1–7) and Fig 3B (lanes 8, 9) respectively. (TIF) [file pone.0148753.s006.tif]

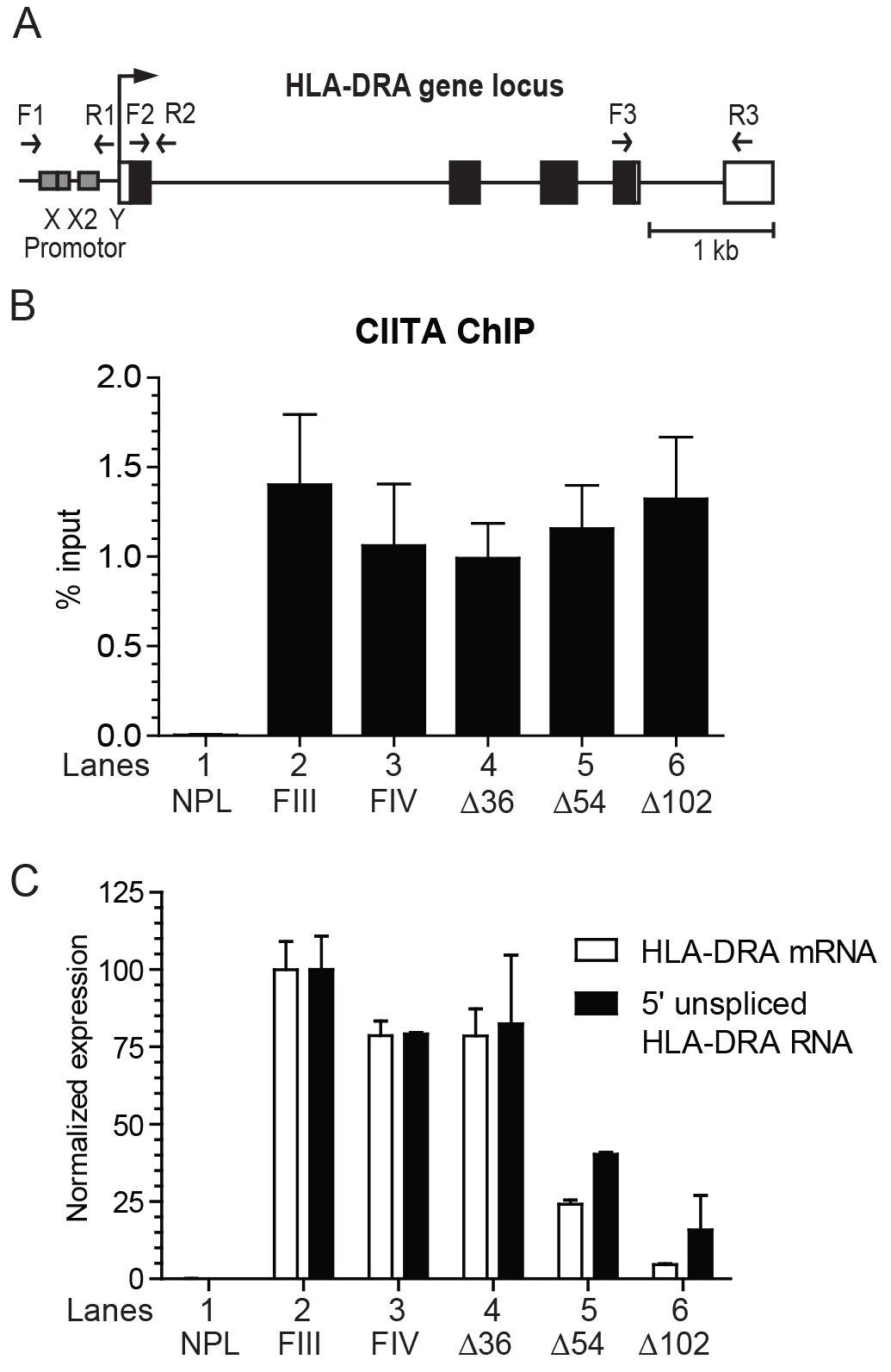

Supplement: S7 Fig — A) Schematic drawing of the HLA-DRA locus and location of primers for gene expression and ChIP analysis. B) CIITA/chromatin complexes from the indicated stable transfectants in HEK293-EBNA cells were immunoprecipitated with the CIITA-specific antiserum K22. The HLA-DRA promoter region was amplified using primers F1 and R1 and measured by QPCR. Values and standard errors are from four independent ChIP experiments. C) Mature (spliced) and unspliced HLA-DRA mRNA expression was determined by RT-QPCR. Absence of DNA contamination was confirmed by absence of amplification with samples not treated with reverse transcriptase (data not shown). Expression levels of CIITA-FIII expressing cells were arbitrarily set at 100. Values are derived from two of the cell preparations used for the ChIP analysis shown in (B). Statistical analysis revealed that differences of the expression levels between mature and unspliced HLA-DRA RNAs for the different constructs are not significant. (TIF) [file pone.0148753.s007.tif]

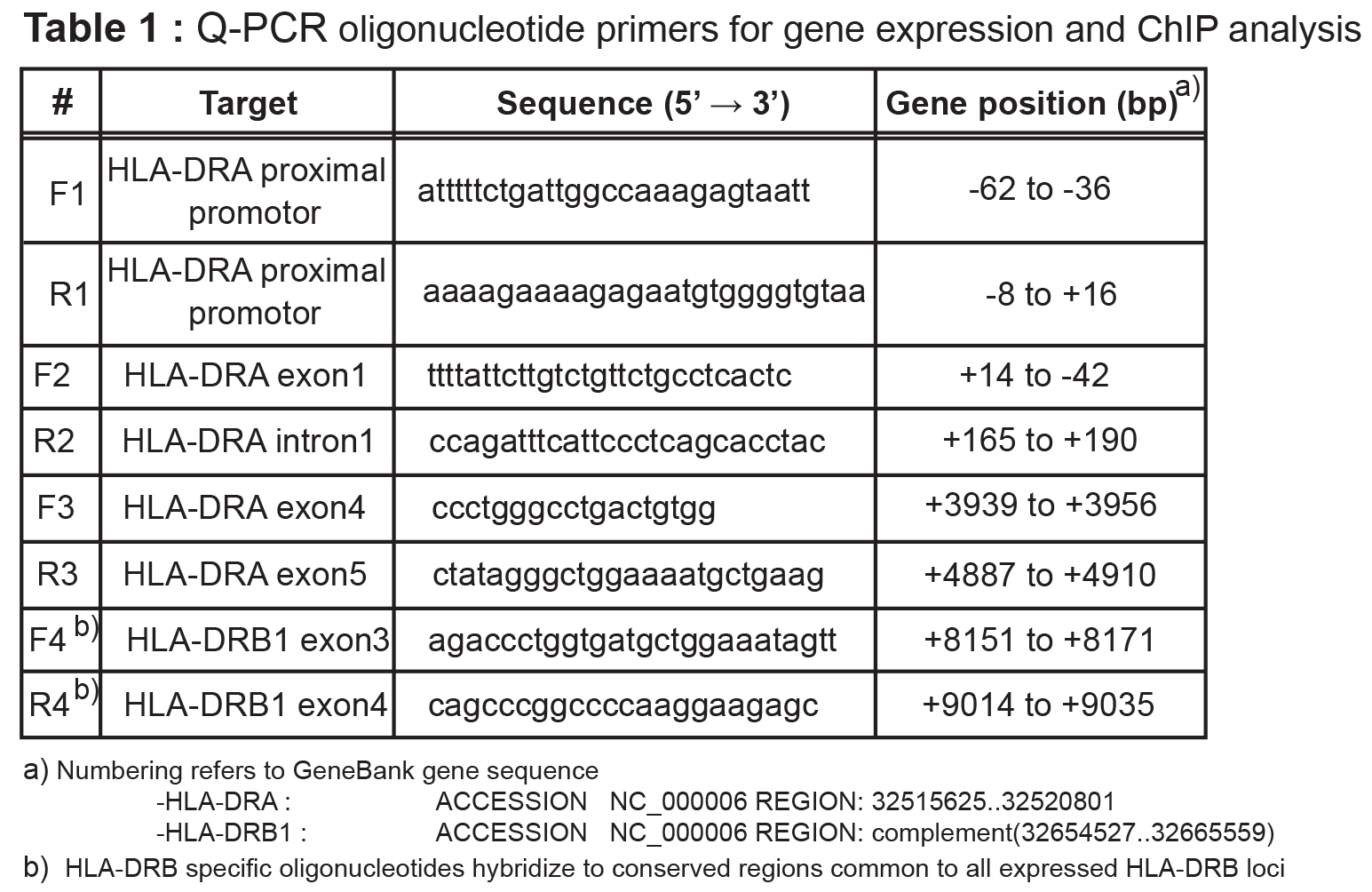

Supplement: S1 Table — (TIF) [file pone.0148753.s009.tif]
