# Supplementary material for: Degradation, Promoter Recruitment and Transactivation Mediated by the Extreme N-Terminus of MHC Class II Transactivator CIITA Isoform III
Source: PLoS One. 2016 Feb 12;11(2):e0148753. doi: 10.1371/journal.pone.0148753 (PMC4752451; doi:10.1371/journal.pone.0148753)
Supplement: S1 Materials and Methods — (DOCX) [file pone.0148753.s008.docx]

**Supplementary Materials and Methods**

**Transient HEK293-EBNA transfections.** HEK293-EBNA cells were transiently transfected by CaPO_4_ transfection in 60 mm cell culture dishes using 500 ng of CIITA-expression vectors, 100 ng of EBS-EGFP and completing to 2.5 µg of DNA with empty EBS-NPL vector. Cells were analyzed by flow cytometry and proteins harvested 3 days after transfection. For determination of HLA-DR expression levels by flow cytometry, cells were gated on EGFP-positive cells.

**Pulse chase analysis.** Pulse chase analysis was carried out as described in [24]. Briefly, Raji and RJ.2.2.5 cells (3 x 10^6^ cells per time point) were starved for 2 h with minimum essential medium without L-methionine (Gibco) and then labeled with 50 mCi/ml (15 µg/ml) L-[^35^S]methionine for 1 h. After the pulse, cells were washed twice and incubated with DMEM medium containing 45 mg/l L-methionine for the indicated times. Cells were recovered by centrifugation and snap frozen. Total protein was isolated by the freeze-thaw method. For immunoprecipitations, 50 µl magnetic beads (sheep anti-rabbit Ig, Dynal) were washed three times with buffer WP-1 (Roche; 50 mM Tris-HCl, pH 7.5, 150 mM NaCl, 0.1% NP-40) and incubated overnight at 4°C with 5 µl CIITA-specific antiserum #21 [27] in 50 µl WP-1. Beads were washed twice and incubated with 50 µl of total protein lysate at 4°C in WP-1 for 3 h. Samples were washed twice in WP-1, twice in WP-2 (Roche; 50 mM Tris-HCl pH 7.5, 500 mM NaCl, 0.1% NP-40) and once in WP-3 (Roche; 50 µM Tris-HCl pH 7.5, 0.1% NP-40). Proteins were resolved by SDS-PAGE and dried gels were exposed to X-ray film (XOMAT AR; Kodak). Quantification was carried out by phosphorimager analysis (Fuji).
